# Spatial ecology of breast cancer reveals co-evolution of proliferative and dormant niches

**DOI:** 10.1186/s13073-026-01711-0

**Published:** 2026-07-09

**Authors:** Cenk Celik, William A. Weston, Thais de Moraes-Lacerda, Eloise Withnell, Shi Pan, Tooki Chu, John Labbadia, Alexis R. Barr, Maria Secrier

**Affiliations:** 1 UCL Genetics Institute, Department of Genetics, Evolution and Environment, University College London, Gower Street, London, WC1E 6BT UK; 2 Cell Cycle Control Team, MRC Laboratory of Medical Sciences, London, W12 0HS UK; 3 NanoCell Interactions Lab, Departamento de Bioquímica E Biologia Tecidual, Instituto de Biologia, Universidade Estadual de Campinas (UNICAMP), Campinas, SP Brazil; 4 Institute of Healthy Ageing, Department of Genetics, Evolution and Environment, University College London, Gower Street, London, WC1E 6BT UK; 5 Institute of Clinical Sciences, Faculty of Medicine, Imperial College London, London, UK

**Keywords:** Breast cancer, Quiescence, G0 persister cells, Spatial transcriptomics, Tumour microenvironment, Tumour dormancy

## Abstract

**Background:**

Cancer progression involves not only uncontrolled proliferation but also the strategic entry of tumour cells into reversible (quiescent) or irreversible (senescent) states of cell cycle arrest (G0). These states can give rise to rare persister-like cancer cells that survive hostile tumour microenvironment conditions, facilitating drug resistance, metastasis and disease relapse. Despite their importance, identifying and understanding the mechanisms regulating these cell populations remains challenging.

**Methods:**

We leveraged single-cell and spatially profiled primary breast tumours to quantify G0 arrest and proliferation decisions in cancer cells, revealing molecular and spatial features associated with proliferation-G0 dynamics.

**Results:**

We uncovered a G0 persister-like state with reduced copy number alteration burden and hallmarks of dormancy, characterised by transcriptional reprogramming of stress response pathways and increased epithelial-mesenchymal plasticity. Spatial analyses revealed distinct ecological niches: G0 cells inhabited protective niches with complement pathway activity proximal to *CXCL10*+ macrophages and myofibroblastic cancer-associated fibroblasts (CAFs), whereas proliferative zones were associated with *CLEC9A*+ dendritic cells and PERK signalling, with distinct drug sensitivities.

**Conclusions:**

Our findings highlight key principles underpinning G0-proliferation dynamics and niche specialisation in breast cancer, offering novel insights into the spatial drivers of tumour heterogeneity and evolution.

**Supplementary Information:**

The online version contains supplementary material available at https://doi.org/10.1186/s13073-026-01711-0.

## Background

The unchecked proliferation of cancer cells is an established hallmark [1], driving tumour evolution and treatment resistance [2, 3]. Proliferation can opportunistically halt if cells exit the cycle in the G0 phase, either reversibly (quiescence, dormancy) [4–6] or irreversibly (senescence) [7], as an adaptive response to internal or environmental pressures. These pauses allow cells to withstand hostile conditions within the tumour microenvironment (TME), thereby promoting drug tolerance and therapeutic persistence [8, 9]. This G0 phenotype may either be an inherent trait of drug-tolerant persister cells or an acquired adaptive state [10, 11], facilitating immune evasion [12, 13] and niche adaptation [14, 15], and fostering minimal residual disease with an increased risk of relapse [11, 16, 17].

Maintaining an equilibrium between proliferation and cell cycle arrest is essential for tumour evolution. Breast cancer is an exemplar where cell cycle dynamics underpin clinical outcomes, with oestrogen receptor (ER)-positive (ER+) subtypes exhibiting slower proliferation but late recurrence through clinical dormancy, while triple-negative breast cancer (TNBC) is marked by aggressively proliferating cells and bears the worst prognosis [18, 19]. Epigenetic mechanisms have been implicated in controlling cell cycle switches in ER+ tumours: epithelial-mesenchymal plasticity helps maintain slow proliferation of disseminated tumour cells and prevents reawakening from dormancy [20, 21], while chromatin remodelling aids dormancy-driven resistance to endocrine therapies [22]. Cell cycle activity is also influenced by interactions with the TME, which further impacts therapy outcomes. For instance, Baldominos et al. [23] describe a quiescent, fibroblast-enriched niche capable of evading immune detection in TNBC.

The clinical relevance of G0 arrest extends beyond breast cancer, with reports of slow-cycling, revival stem cell phenotypes linked with poor prognosis and chemoresistance in colorectal cancer [24–26], or quiescent *PROM1*+ cells implicated in recurrence and resistance in paediatric high-grade gliomas [27]. However, studying G0 arrested cells remains challenging due to their diverse phenotypes [28, 29], scarcity within tumours and plastic switching between proliferative and quiescent states. The often short-lived nature of this state further complicates its identification, and the signalling pathways controlling G0 arrest are still poorly understood. Additionally, the interplay between intrinsic factors [30, 31] and the tumour ecosystem in shaping cancer evolution is crucial in determining invasiveness [32] and therapy resistance [33–35], but remains inadequately explored.

We previously developed a method derived from a quiescence signature of non-transformed cells to quantify G0 arrest in tumours using bulk transcriptomic data, identifying genetic regulators like CEP89 that modulate the capacity for G0 entry [36]. Here, we derive a tumour-specific G0 arrest signature in single cells, validate it experimentally and employ it to elucidate how G0 arrest and proliferation dynamics shape breast cancer ecosystems and evolutionary adaptation at single-cell and spatial resolution. We identify a G0 persister-like state characterised by reduced copy number alteration burden, integrated stress response (ISR) and hybrid epithelial-mesenchymal features. We uncover spatially segregated G0 arrest and proliferative niches with distinct immune associations, with G0 arrested spots occupying protective macrophage-proximal niches and displaying differential drug sensitivities. Finally, we develop a foundation model to identify cell proliferation and G0 states at single-cell resolution in breast cancer, available at https://github.com/secrierlab/G0-FM. Our findings offer insights into how balancing proliferation and G0 arrest drives tumour adaptation and phenotypic diversity.

## Methods

### Study cohorts and datasets

Single-cell RNA-seq data were obtained from the Curated Cancer Cell Atlas of the Weizmann Institute of Science, encompassing three studies: Gao et al. [37], Pal et al. [38] and Qian et al. [39]. The integrated dataset comprised 138,727 cells from 43 breast cancer patients spanning ER+ (*n* = 17), TNBC (*n* = 11), Her2+ (*n* = 6), PR+ (*n* = 1), DCIS (*n* = 1) and neoplastic (*n* = 7) subtypes, together with four healthy individuals. Of these, 91,897 epithelial cells (85,749 malignant; 6,148 normal) were used for cell cycle state classification, gene regulatory network inference, proteostasis analysis and ligand–receptor communication analyses. The tumour-specific G0 arrest signature was derived from the dataset of Oren et al. [40] (GEO: GSE150949).

Twelve 10x Visium slides from breast cancer tissues were sourced from Barkley et al. [41] (slides 0–2), 10x Genomics (slides 3–5) [42] and Wu et al. [35] (slides 6–12), covering Luminal A (*n* = 4), Luminal B (*n* = 2), Her2+ (*n* = 2) and TNBC (*n* = 4) subtypes. A total of 32,845 spots were analysed. These data were used for hotspot mapping, spatial niche characterisation, ligand-receptor analysis and drug-niche interaction scoring. Single-cell resolution Xenium data were obtained from Janesick et al. [43] via 10x Genomics and used for orthogonal validation of spatial proximity relationships between G0 cells and *CXCL10*+ macrophages/myCAFs.

Bulk RNA-seq and gene-level copy number data from breast cancer primary tumours were obtained from the The Cancer Genome Atlas (TCGA) Pan-Cancer Atlas. Metastatic tumour data were obtained from the MET500 cohort [44]. Both were used for logistic regression modelling of genomic drivers of G0 arrest versus proliferation. Relapse-free survival analyses were performed on the METABRIC cohort, stratified by G0 score.

Eight breast cancer cell lines were used for validation of the G0 scoring signature: MCF7, BT474, T47D and ZR751 (ER+/Luminal) and HCC1937, HS578T, MDAMB168 and MDAMB231 (TNBC/Basal). Quiescent fractions were quantified by EdU incorporation assays with three biological replicates per line. MCF7 cells with endogenously labelled mRuby-PCNA were used for timelapse imaging validation of cell cycle state transitions.

### Data integration and cell type annotation

To preprocess and integrate the single-cell samples, the standard Seurat pipeline with SCTransform() v2 normalisation [45] was employed, wherein low-quality cells (with mitochondrial transcript percentage > 20) were removed, and cell cycle phases were regressed out. Subsequently, the Leiden community detection algorithm was applied with a resolution of 1.2 on the 29 principal components of the integrated data, followed by uniform manifold approximation projection (UMAP) for dimension reduction.

Cell type annotation was performed at two resolutions. Initially, main cell types were identified using cell type signatures from PanglaoDB [46] and *ScType* R package [47]. Then, for further refinement, the CELL type iDentification tool on the Deeply Integrated Human Single-Cell Omics platform [48] was employed, by utilising averaged expressions of Leiden communities to accurately annotate cell types. Additionally, the top two differentially expressed genes of the annotated Leiden communities were calculated on the integrated data with a log2 fold-change of 0.25 and minimum percentage of 0.25 using the likelihood-ratio test for single-cell feature expression [49].

To identify malignant cells within the epithelial cluster, the *inferCNV* R tool [50] was utilised to infer copy number alterations (CNAs) from gene expression data. InferCNV was configured with the following parameters: a cutoff of 0.1, deemed suitable for 3’ sequencing droplet-based assays, denoising = TRUE, HMM_type = “i6” for Hidden Markov Model (HMM)-based CNA prediction, and analysis_mode = “subclusters” to identify clonal CNAs. A heat map depicting the relative expression intensities across each chromosome was generated, revealing regions of the tumour genome that were over- or under-abundant compared to normal cells.

### Differential gene expression analysis

We conducted differential gene expression analysis on the integrated dataset using Seurat’s FindAllMarkers() function on the RNA layer of the Seurat object with parameters min.pct set to 0.5 and logfc.threshold of 1 for only positively upregulated genes for both cell type annotations and cell cycle states.

### Quantification of G0 arrest in malignant single cells

First, based on inferred CNAs, the malignant cells were subsetted to implement a combined z-score accounting for genes upregulated and downregulated in quiescence, as previously described by us in Wiecek et al. [36]. We derived a tumour-specific G0 arrest signature from epithelial tumours that were demonstrably non-cycling, using the dataset of Oren et al. [40]. Differential expression analysis between non-cycling and cycling tumour epithelial cells yielded tumour-specific upregulated and downregulated G0 gene sets. Applying this revised signature restored biological concordance, producing a strong positive correlation between the G0 score and experimentally measured quiescent fractions in cell lines (Spearman *r* = 0.79; Fig. 2a). Z scores of the upregulated and downregulated genes in the G0 arrest signature were then calculated using the GSVA R package. Next, a final G0 arrest score was obtained by subtracting the two scores. Finally, the G0 arrest scores were used for the discretisation of malignant cells into three categories: (1) G0 persister cells, (2) cycling and (3) intermediate. Cut-off values were calibrated by maximising the inverse correlation between negative G0 scores and an independent proxy for EdU incorporation based on known S-phase targets. The same strategy was applied to define thresholds for the G0+ population. This approach ensures that selected cut-offs are constrained not only by statistical extremity but by alignment with independent biological proxies of cell cycle activity, strengthening confidence that G0-classified cells represent a *bona fide* non-cycling state. The tumour-specific G0 signature differs substantially from the non-malignant quiescence signature used in our earlier work [36]. Whereas the original signature, derived from cultured non-malignant cells [51], contained genes associated with RNA processing and splicing, nuclear transport, SUMOylation, genome maintenance, stress response and cell cycle control, the tumour-specific signature was derived from non-cycling tumour epithelial cells and captures arrest programmes shaped by oncogenic and microenvironmental stress, including immune modulators (e.g. *HLA-A*, *HLA-B*), ECM remodellers (e.g. *TACSTD2*, *LGALS3*) in the upregulated set, alongside canonical proliferation markers in the downregulated set. A full comparison of the two gene sets and enriched pathways from ConsensusPathDB are provided in Table S2.

### Genomic alterations in primary tumour cells

Copy number alterations (CNAs) were inferred from single-cell RNA-seq data using inferCNV (version 1.8.1) [50]. Non-malignant cells were used as the reference population to estimate baseline expression levels. CNA profiles were generated across chromosomal regions, with amplifications and deletions visualised as positive and negative deviations from baseline, respectively (Fig. 3a). CNA burden per cell was quantified as the sum of absolute CNA z-scores across all genomic regions for each cell. CNA burden was compared across cell cycle states (cycling, intermediate and G0 arrested) using the Wilcoxon rank-sum test.

Intratumoral heterogeneity based on copy number alterations (ITH-CNA) was calculated as described in Wu et al. [35] separately for each patient and cell-cycle state using inferred CNA profiles generated by inferCNV. For each patient-state combination, a cell-by-cell Pearson correlation matrix was computed from the inferred CNA profiles. ITH-CNA was defined as the interquartile range (IQR) of the resulting distribution of Pearson correlation coefficients:

$${R}_{ij}=\mathrm{c}\mathrm{o}\mathrm{r}\left({C}_{i},{C}_{j}\right)$$

$$IT{H}_{ps}={Q}_{75}\left({R}_{ps}\right)-{Q}_{25}\left({R}_{ps}\right)$$

where ${C}_{i}$ and ${C}_{j}$ denote the CNA profiles of cells *i* and *j*, respectively, and ${R}_{ps}$ represents the correlation matrix for patient *p* and cell cycle state *s*. Cell cycle states containing fewer than two cells within a patient were excluded. To account for differences in overall CNA heterogeneity between patients, ITH-CNA values were standardised within each patient across cell-cycle states using z-score transformation:

$${z}_{ps}=\frac{IT{H}_{ps}-{\mu}_{p}}{{\sigma}_{p}}$$

where ${{\boldsymbol{\mu}}}_{{\boldsymbol{p}}}$ and ${{\boldsymbol{\sigma}}}_{{\boldsymbol{p}}}$ are the mean and standard deviation of ITH-CNA values across all cell cycle states within patient *p*. The resulting patient-level z-scores were compared between cell cycle states using two-sided Mann–Whitney U test.

### Cell culture

MCF7, BT474, T47D, ZR751 cell lines were maintained in DMEM (Gibco) supplemented with 10% FBS (Sigma) and 1% Penicillin–Streptomycin (P/S: Gibco). HCC1937, HS578T, MDAMB168 and MDAMB231 cell lines were maintained in RPMI-1640 (Gibco) supplemented with 10% FBS (Sigma), 2 mM Glutamine and 1% Penicillin–Streptomycin. All cells were grown at 37ºC and 5% CO_2_ in a humidified incubator. Cell lines were maintained at a passage between P6 and P12 and were frequently tested for mycoplasma contamination.

### Quiescent fraction estimation in cell lines

All cell lines were seeded at 1000 cells/well in 384-well Phenoplates (Revvity) in 20 µl of cell media then incubated for 24 h to attach to the plate and begin proliferating. Cell culture media was supplemented with EdU to a final concentration of 10 µM for 24 h, then fixed at room temperature (RT) in 4% PFA (Thermo Fisher) for 10 min. Fixed cells were permeabilised with 0.1% Triton solution in PBS for 15 min, then incorporated EdU was stained using the Click-IT EdU imaging kit (Invitrogen), according to the manufacturer’s instructions. All nuclei were then stained using 1 µg/ml Hoechst 33258 (Sigma) for 10 min at RT. Cells were kept in PBS prior to imaging.

All fixed cells were imaged on an Operetta CLS High-Content Analysis System (Revvity) with a 20X (N. A. 0.8) objective. Quantitative image analysis was performed using Harmony High-Content Imaging and Analysis Software (Revvity). To segment nuclei: nuclei were segmented based on Hoechst intensity. Nuclei at the edge of the field of view or less than 70 µm^2^ were excluded to remove partially segmented and dead cells. Measuring EdU signal intensities: once segmented, Harmony software was used to automatically calculate the mean intensity of EdU signal in the defined nuclear or nuclear ring region. EdU classification: cells were classified into EdU- or EdU+ based on their EdU ratio. The EdU ratio was calculated based on the EdU signal intensity within the nuclear region and the nuclear ring region, a 2µm^2^ wide perimeter outside the nucleus. Nuclear EdU/Ring region (cytoplasmic) EdU = EdU ratio. Cells with an EdU ratio < 1.2 were classified as EdU- and thus quiescent, while those > 1.2 were EdU+.

### Generating MCF7 mRuby-PCNA cells

MCF7 cells with endogenously labelled mRuby-PCNA were generated as previously described [52]. After viral transduction, FACS was used to sort mRuby-expressing cells to single cell density in 96-well plates containing 50:50 full:conditioned media. Cells were expanded and then checked for mRuby-PCNA expression on the Operetta CLS High Content Analysis System microscope (Revvity).

### Timelapse imaging and analysis

Live cell imaging was performed using MCF7 mRuby-PCNA cells cultured in 384-well Phenoplates (Revvity) with a breathable film sheet to prevent media evaporation. Cells were plated at a density of 500 cells/well 24 h prior to imaging. Cells were imaged using the Operetta CLS High Content Analysis System microscope (Revvity), with atmospheric conditions kept at 37°C and 5% CO_2_. Imaging was performed with a 20X (N. A. 0.8) objective at 10-min intervals for 72 h, using the Alexa-568 channel for mRuby-PCNA detection. Images were exported from the Harmony High-Content Imaging and Analysis Software (Revvity) and arranged into stacks in Fiji (ImageJ). Individual cells were tracked by eye across stacks to determine cell cycle phases changes.

### Genomic associations with G0 arrest/proliferation in single cells and large cancer cohorts

Gene-level copy number changes called by inferCNV in the single cell data were compared between G0 persisters and cycling cell populations, and changes significantly enriched in either category were identified using a Fisher’s exact test, with Benjamini–Hochberg adjustment of p-values for multiple testing.

RNA sequencing data and gene-level copy number data from primary tumours from the TCGA Pan-Cancer Atlas were downloaded using *TCGAbiolinks* R package [53]. FPKM-normalised RNA-sequencing data and segmented copy number data from metastatic tumours were downloaded from the MAGI MET500 server (https://met500.med.umich.edu/downloadMet500DataSets), along with processed data on recurrent molecular aberrations from the MET500 study [44]. To map copy number segments to genes, the *GenomicRanges* R package was used to convert segment data into genomic ranges, which were annotated with gene-level data using the *annotatr* R package. A list of 723 known cancer driver genes was sourced from the COSMIC database. Each sample was assigned a G0 score as described above. Tumours with a G0 score above 1.5 were classified as G0 arrested, while those with scores below −1.5 were considered to be cycling. Logistic regression was employed to predict high levels of proliferation versus G0 arrest in TCGA and MET500 cohorts, with model selection performed using the stepAIC() function from the *MASS* R package. For model building, samples were split into training (75%) and testing (25%) datasets using the createDataPartition() function. A 10-fold cross-validation procedure was implemented using the trainControl() function from the *caret* R package. The logistic regression model trained on the MET500 dataset achieved an accuracy of 62.5% with an AUC of 0.66, whilst the model trained on TCGA dataset has an accuracy of 72.7%, with an AUC of 0.78.

### Gene set enrichment analysis

Hallmark gene set enrichment analysis on single cells was performed using the *decoupler* package. The epithelial-mesenchymal transition (EMT) score was calculated as described in Malagoli-Tagliazucchi et al. [54]. Endoplasmic reticulum stress-related pathways scores were computed using the *irGSEA* R package [55], with curated gene signature lists as input. Dormancy signatures were taken from Ren et al. [55], Ruth et al. [56], Cheng et al. [57], Kim et al. [58] and Montagner et al. [59].

### Spatially coherent enrichment analysis

G0 score signatures within tumour spots were calculated with our EnrichMap framework [60] in Visium slides, with n neighbourhood of six. The thresholds for the cycling states were determined as described above in single cells. For demonstrating the ligand-receptor pairs spatially, number of neighbours was set to 24 to ensure long distance effects of ligands.

### Cell–cell interactions

TME-receiver cell interactions were assessed by two methods:

1. Estimation of putative ligands and downstream genes in the receiver population.
  The estimation of ligands in the TME potentially driving gene expression in G0 arrest or cycling cells involved utilising *NicheNet* v2 R package [61]. Differentially expressed genes with a log2 fold-change greater than 0.5 and an adjusted *p*-value lesser than 0.05 were considered for ligand prioritisation in the receiver population. Background genes were defined as genes expressed in the receiver population (at least 5% of the cells). Ligands were not constrained to be expressed solely by putative sender cells within the dataset, allowing for the assessment of TME ligands produced by non-immune cells such as tumour cells or the stromal compartment. The prioritisation of ligands was based on the area under the precision-recall (AUPR) scores, indicating whether a gene significantly correlated with their respective ligand-target regulatory potential. The ligand-target regulatory potential reflects the strength of association between a ligand and a target gene based on prior knowledge, presented in NicheNet’s integrated weighted networks.
2. Estimation of ligand-receptor pairs:
  To infer ligand-receptor pairs between the TME and receiver cells regardless of their effects on downstream gene expression, the differentially expressed interaction module of CellPhoneDB [62] was employed, with a threshold of 0.1 percent gene expression in each cell. This method utilises a list of differentially expressed genes sorted by adjusted p-values calculated by Seurat’s FindAllMarkers() function for each cell type.
  In spatial transcriptomics, we applied the LIANA+ package’s CellPhoneDB module [63] to identify spatially relevant ligand-receptor pairs.

### Gene regulatory networks

Single-Cell rEgulatory Network Inference and Clustering Python implementation (pySCENIC) [64] was employed to infer gene regulatory networks (GRNs) from single cells across G0 arrested, cycling and intermediate tumours. The inference of gene regulatory networks was based on *hg38_v10nr_clust *databases, focusing on regions around 10 kb, 100 bp downstream, and 500 bp upstream of transcription start sites (TSS), using score and ranking matrices provided by the SCENIC pipeline.

The top 50 GRNs were grouped by cancer subtype for AUCell scoring, and Spearman correlation was applied to identify conserved GRN modules. Binding motifs for selected transcription factors were inferred from the *motifs-v10nr_clust-nr.hgnc-m0.001-o0.0.tbl* database.

### Gene expression module analysis

Gene expression modules were identified using Hotspot [65]. For each cell cycle state (G0, cycling and intermediate), malignant cells were analysed separately to identify state-specific gene expression programmes. A cell similarity graph was constructed based on transcriptional similarity using the top principal components. The depth-adjusted negative binomial (DANB) model was used to fit per-cell expectations for each gene, accounting for differences in sequencing depth. Feature selection was performed to identify genes exhibiting non-random patterns of expression within the similarity graph based on local autocorrelation statistics, retaining genes with significant autocorrelation (FDR < 0.05). Pairwise local correlations were computed between selected genes to construct a Z-score matrix conditioned on the cell similarity graph structure, and hierarchical clustering was applied to group genes into modules. Module scores were computed per cell and visualised as heatmaps showing the Z-score of module activity across cells. Functional annotation of modules was performed based on enriched biological processes within each gene set.

### Spatial transcriptomics analyses

Breast cancer Visium slides were sourced from Barkley et al. [41] (slides 0–2), 10X Genomics [42] (slides 3–5) and Wu et al. [35] (slides 6–12). These data sets were merged into a unified AnnData python format for analysis. Pre-processing, normalisation and cell type deconvolution were carried out as conducted in previous studies [66]. A total of 32,845 spatially profiled spots were analysed. Spots were retained if they showed at least 100 genes with a minimum count of 1 per cell, had over 250 counts per spot, and less than 20% mitochondrial counts per cell. Cellular deconvolution was performed using cell2location [67] with a scRNA-seq breast cancer dataset from Wu et al. [35], comprising 100,064 cells from 26 patients across 21 cell types. These included cancer epithelial cells (basal, cycling, Her2+, Luminal A, Luminal B), B cells (naïve and memory), CAF subtypes (myCAF-like and iCAF-like), perivascular-like cells (immature, cycling, differentiated), T cells (cycling, CD4, CD8 T cells), cycling myeloid cells, dendritic cells, endothelial cells (*ACKR1*, *CXCL12*, *RGS5*), *LYVE1*-expressing lymphatic endothelial cells, luminal progenitors, mature luminal cells, macrophages, monocytes, myoepithelial cells, NK cells, NKT cells and plasmablasts. Tumour cells in the spatial transcriptomics dataset were identified using the STARCH Python package, which infers copy number alterations [68].

Hotspot analysis was conducted using our SpottedPy framework [66], where the gene signatures were scored using Scanpy’s score_genes() function. G0 and cycling tumour spots were calculated using the gene sets described as above.

### Drug-niche interactions

To interrogate drug-niche interactions, we utilised drug2cell [69] to score candidate drugs and their targets with differential correlations to drug sensitivity by leveraging gene expressions in each cell state, then employed our SpottedPy [66] method to evaluate the spatial distances between drug niches and cell cycle states.

### Ki67 expression prediction and validation in spatial transcriptomics

To validate the alignment between the G0 score and established proliferation markers, we predicted Ki67 (*MKI67*) expression status in matched Visium spatial transcriptomics and H&E slides using a deep learning approach [70]. Spots were classified as Ki67+ or Ki67- based on *MKI67* expression levels, with spots exceeding a defined expression threshold designated as Ki67+. The G0 score was calculated for each spot as described above, and the spatial distribution of Ki67 status and G0 scores were visualised across tissue sections from ER+ (Luminal A and Luminal B), Her2+ and TNBC (Basal-like) subtypes. Per-slide Ki67+ density was calculated as the proportion of Ki67+ spots among all tumour spots. The correlation between mean G0 score and Ki67+ density was assessed using Spearman's rank correlation across slides (Fig. S10b). Statistical significance was determined with a two-tailed test.

### The single-cell foundation model

The G0-FM model is an advanced architecture comprising three specialised networks, adapted from the scBERT framework [71] to address the specific challenges of G0 arrest in single-cell analysis. Its two-stage training pipeline bridges the gap between large pre-trained datasets and the specialised requirements of G0 arrest data [72]. In the first stage, the model’s embedding space is refined using a Low-Rank Adaptation (LoRA) approach [73], a Parameter Efficient Fine-Tuning (PEFT) strategy that enables adaptation to scRNA-seq data while reducing overfitting risks.

The G0-FM architecture follows that of EMT-FM [72] and incorporates a multiplication layer that leverages fine-tuned language models to amplify signals from highly expressed genes. Attention vectors generated by these models act as weighted enhancements of raw gene expression data, selectively highlighting relevant cellular features. To optimise model focus, genes with low expression or peripheral relevance to the EMT process are systematically filtered, concentrating learning on critical pathways. For classification, a Multilayer Perceptron (MLP) serves as the final module to yield predictions. Ultimately, this fusion network provides a computational model of cell states under G0 arrest, distilling complex gene expression profiles into accurate cell state outputs.

### Statistical analysis

Groups were compared using a two-sided Student’s *t*-test, Wilcoxon rank-sum test, ANOVA or Kruskal–Wallis test, as appropriate. Adjustments for multiple testing were made using the Benjamini–Hochberg method when necessary. False discovery rates (FDR) were calculated by taking the -log10 of the adjusted *p* values. Graphs were generated using either the *ggplot2* and *ggpubr* R packages or the *matplotlib* and *seaborn* python libraries.

## Results

### G0 arrest is a frequent state with prognostic relevance in primary breast cancer

**Fig. 1:**
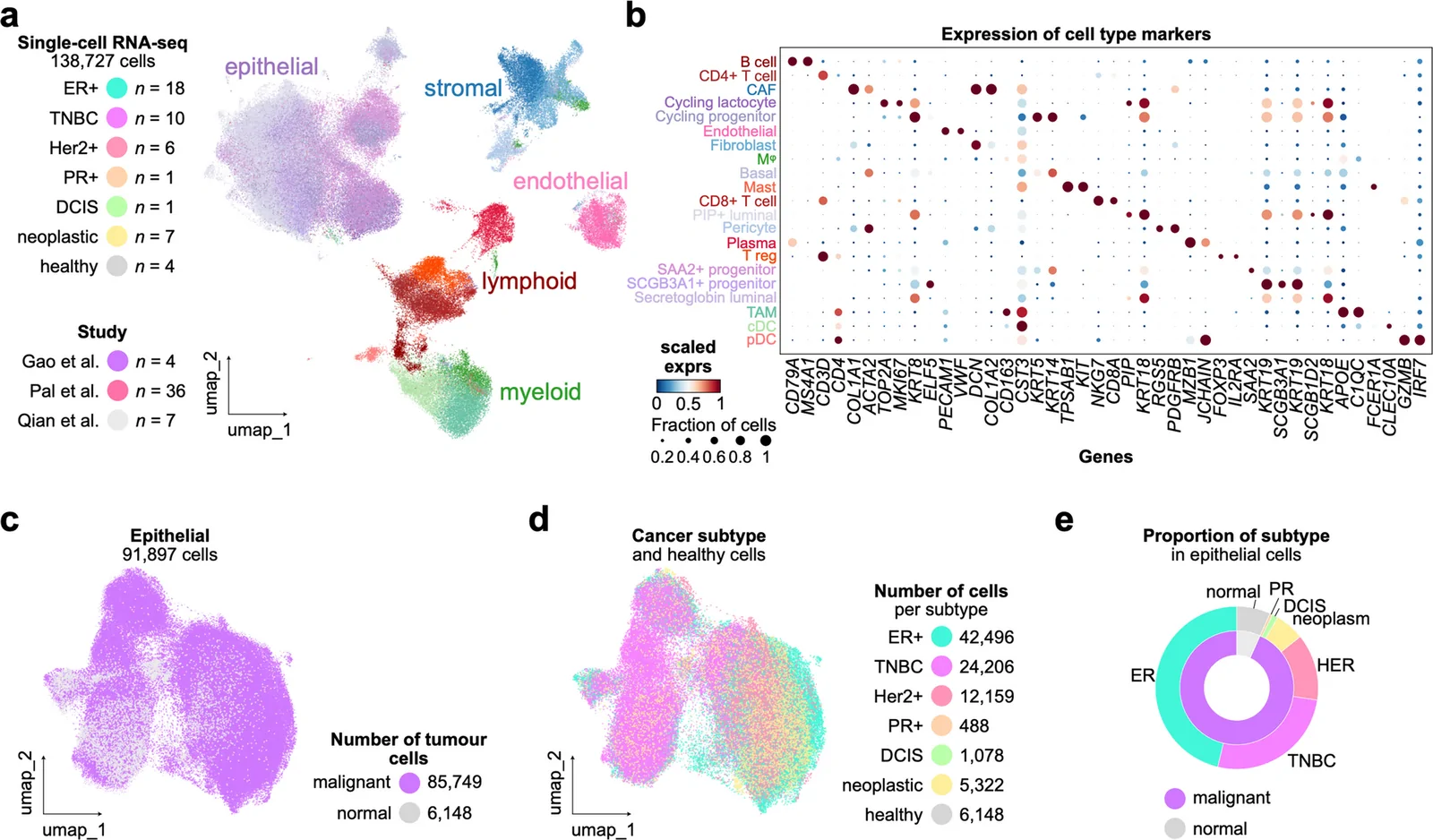
Single-cell primary breast cancer transcriptomic atlas. **a** UMAP visualisation of 138,727 cells from single-cell RNA sequencing of breast cancer samples, coloured by major cell lineages. Sample composition by cancer subtype (ER+, *n* = 17; TNBC, *n* = 11; Her2+, *n* = 6; PR+, *n* = 1; DCIS, *n* = 1; neoplastic, *n* = 7; healthy, *n* = 4) is indicated. **b** Dot plot showing expression of cell type marker genes. **c** UMAP of 91,897 epithelial cells, distinguishing malignant (*n* = 85,749) from normal (*n* = 6,148) cells, inferred by inferCNV. **d** UMAP of epithelial cells coloured by cancer subtype and healthy status, with cell counts per subtype indicated. **e** Donut chart showing proportion of molecular subtypes within malignant epithelial cells

We collated 138,727 cells across breast cancer subtypes, leveraging single-cell RNA sequencing (scRNA-seq) datasets from three studies [37–39] deposited at the Curated Cancer Cell Atlas of the Weizmann Institute of Science [74]. Our atlas covered both non-invasive (including ductal carcinoma *in situ* [DCIS] and neoplastic) and invasive breast cancers—hormone-positive (ER+ or PR+), Her2+ and triple-negative breast cancer (TNBC)—from 43 patients, and four healthy individuals (Fig. 1a-e). A heterogeneous cellular landscape was observed across these tumours and in healthy tissues, including epithelial, fibroblast, myeloid, lymphoid and endothelial cells, collectively representing predominant cell types within the breast cancer ecosystem (Fig. 1a; Table S1). Malignant cells were ascertained as harbouring genomic alterations using inferCNV [50] (Fig. 1c, Fig. S1a-b).

**Fig. 2:**
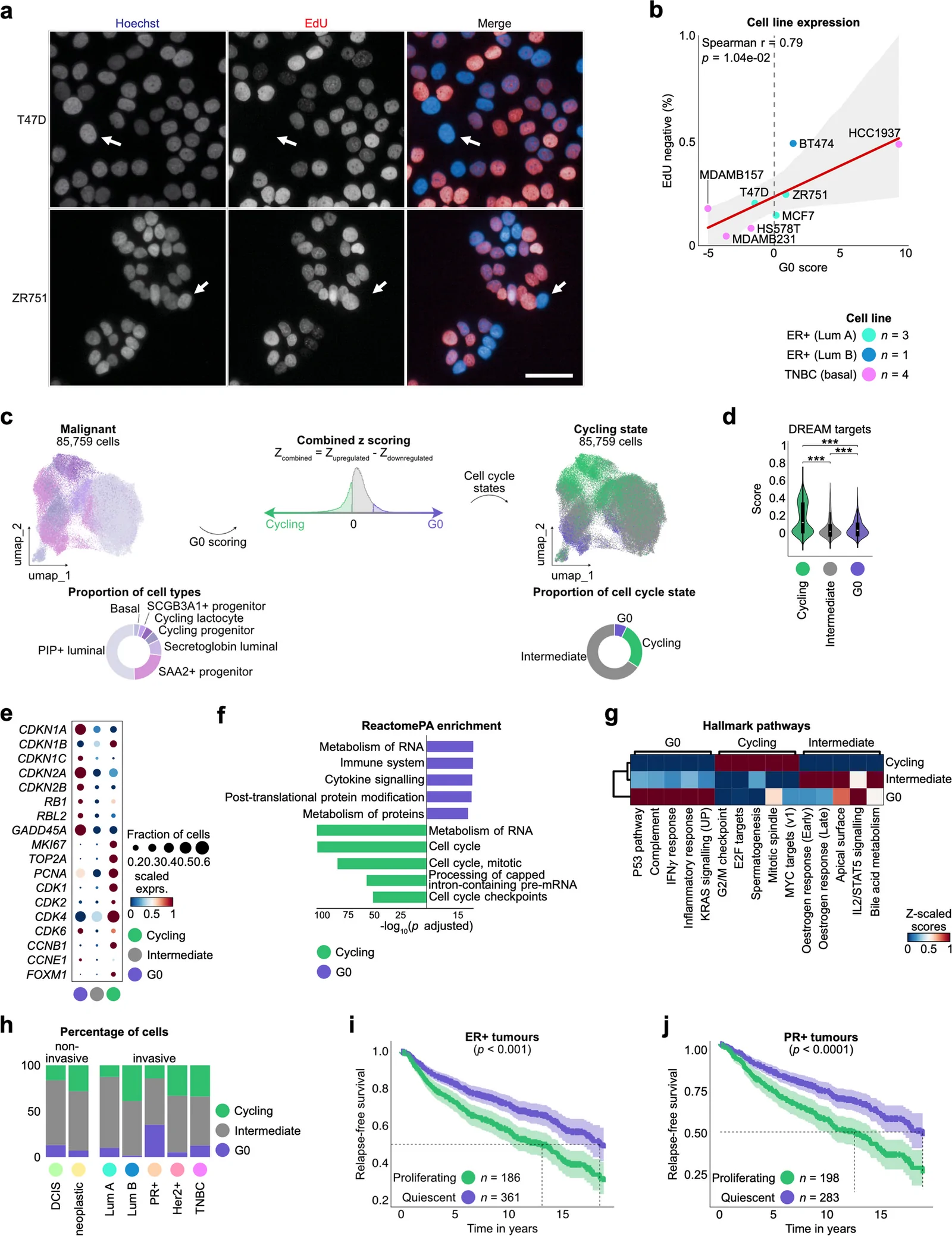
Cell cycle state classification. **a** Images show T47D and ZR751 breast cancer cell lines labelled with EdU for 24 h before fixing and imaging. White arrows point to examples of EdU negative (quiescent) cells. Hoechst is in blue and EdU is in red in the merged images. Scale bar is 50 µm. **b** Validation of tumour-specific G0 scoring signature using bulk RNA-seq from breast cancer cell lines with experimentally quantified quiescent fractions (EdU negative %). Spearman correlation (*r* = 0.79, *p* = 1.04e-02) demonstrates concordance between G0 arrest score and measured quiescence across ER+ (Luminal A, *n* = 3; Luminal B, *n* = 1) and TNBC (basal, *n* = 4) cell lines. Three biological repeats were performed for EdU quantification. **c** Workflow for calculating G0 scores, with UMAP embedding showing malignant cells annotated by discrete cell cycle states. **d** DREAM complex target scores across cell cycle states. ***p < 0.001, Kruskal–Wallis test. **e** Expression comparison of quiescence and proliferation markers across different cell cycle states. **f** ReactomePA enrichment analysis for cycling (green) and G0 (purple) cells, showing top enriched pathways. **g** Hallmark pathway enrichment analysis across cell cycle states. **h** Percentage of cycling, intermediate and G0 cells across non-invasive and invasive disease stages. **i-j** Kaplan–Meier survival plots for relapse-free survival in ER+ (**i**) and PR+ tumours (**j**) from the METABRIC cohort, stratified using the G0 score into quiescent (high levels of G0 arrest) and proliferating (high levels of cycling cells)

We questioned whether long-lived quiescent states akin to those observed in drug-tolerant persister cells (DTPs) may emerge spontaneously in primary tumours before treatment, thereby constituting a potential latent reservoir for therapeutic resistance and metastatic spread. To address this, we defined a transcriptional signature of non-cycling persister cells derived from epithelial cancer cells that underwent sustained proliferative arrest following cytostatic treatment, as described in [40] (see Methods). To assess whether this “*persister*” signature could capture a quiescent state in breast cancer, we validated it across eight breast cancer cell lines, spanning ER+ (Luminal A and Luminal B) and TNBC (basal-like) lines. Comparing persister signature scores inferred from RNA-seq data in these cell lines with their *in vitro* quiescence fractions quantified by lack of 5-Ethynyl-2'-deoxyuridine (EdU) incorporation over a 24-h period [75] revealed a strong positive correlation (Spearman *r* = 0.79, *p* = 0.0104), providing confidence that the persister signature can capture an arrest state across different breast cancer subtypes (Fig. 2a-b, Fig. S2a). We also used timelapse imaging to validate our fixed cell quiescent fraction measurements. We engineered MCF7 cells to express endogenously tagged mRuby-PCNA that allows us to track progression through every cell cycle phase (see Methods) [52]. We imaged cells over a 72-h period and analysed the amount of time spent in each cell cycle stage (Fig. S2b-c). Over the imaging period, we captured 288 G0/G1 phases, of which 26 (11.6%) were longer than 24 h, consistent with our fixed cell imaging data (Fig. 2b).

We next applied this signature to the patient-derived single-cell resolved primary breast cancer dataset, deriving a “*persister*”-like arrest z-score per cell capturing a continuum of cell cycle activity across malignant cells (see Methods; Fig. 2c, Fig. S2e). To capture discrete states aligned with those observed experimentally, we employed a proxy for EdU incorporation in the form of a transcriptional S-phase gene target signature and identified z-score thresholds that maximised negative and positive correlations between this EdU activity proxy and the persistent arrest scores (Fig. S2d). This approach yielded three populations: cycling (27%), intermediate (65.8%) and non-cycling “*persister*”-like (7.2%) cells (Fig. 2c; Table S2). The targets of the transcriptionally repressive DREAM complex, the main effector of quiescence, were expressed in cycling cells but not in the intermediate and “*persister*”-like cells (Fig. 2d). The same was observed for *MKI67* (Fig. 2e), reinforcing that our classification captures cell cycle exit rather than a generic low-proliferation state. Expression of *CDKN1A* (p21) and *RBL2* (p130), canonical mediators of G0 arrest, was markedly elevated in the non-cycling “*persister*”-like cells relative to both the intermediate and cycling populations (Fig. 2e), further supporting that this state is most likely to mark *bona fide* G0, and potentially a deeper form of arrest that is actively maintained, compared to the intermediate population.

These populations bore further expected proliferation and quiescence hallmarks, including cell cycle checkpoint and MYC/E2F target expression in cycling cells and upregulation of p53 signalling in G0 persisters (Fig. 2f-g). Interestingly, the G0 cells also activated the complement and interferon gamma response pathways, while the intermediate cells showed markers of a differentiated luminal/apical phenotype (Fig. 2g). Differential gene expression analysis further confirmed state-specific markers: cycling cells expressed genes associated with microtubule dynamics (*STMN1*, *TUBA1B*), chromatin remodelling (*HMGB2*), cell cycle progression (*CKS1B*) and apoptosis inhibition (*BIRC5*), whereas G0 cells showed elevated expression of immune modulators (*HLA-A*, *HLA-B*) and epithelial adhesion molecules (*TACSTD2*, *LGALS3*) (Fig. S2f).

We also ensured that our scoring method did not mistakenly capture low-quality cells [76], as indicated by *MALAT1* expression (Fig. S2h), nor a senescent state, as all malignant cells lacked senescence markers such as β-galactosidase (*GLB1*; Fig. S2i).

We further validated our classification with ccAFv2 [77], an independent cell cycle classifier that includes a quiescent G0 (qG0) state trained on human neural stem cell data (U5-hNSC). The predicted phase distribution showed most cells in G1 (64.7%), with smaller proportions across S, G2 and M phases (Fig. S2g). Cycling cells were enriched for S/G2/M phases, while the G0 persister cells showed an increased proportion of qG0 assignments compared to cycling and intermediate populations, providing independent support for our G0 classification. However, the qG0 state in ccAFv2 reflects a neural stem cell quiescence programme and does not fully capture the transcriptional and ecological features of breast tumour G0 arrest, limiting its biological correspondence in this context.

Inevitably, the prevalence of these states should align with classical molecular subtypes of breast cancer. Indeed, the Luminal A tumours presented a higher fraction of slower growth (G0 persisters and intermediate cells), whilst cycling cells were more abundant in Luminal B and TNBC samples (Fig. 2h), consistent with the clinical features of these subtypes [18, 19]. Importantly, although G0 persister cells were sometimes a minority, they were nevertheless present across all subtypes and patients (Fig. 2h, Fig. S2j), highlighting that this state is widespread in primary tumours. Notably, TNBC tumours had a high fraction of G0 persister cells across most patients, which may represent a previously underappreciated contributor to their heterogeneity and aggressiveness. Patients with faster proliferating ER+ tumours had shorter relapse-free survival (RFS), whereas those with higher G0 arrest levels manifested longer latency before relapse (Fig. 2i), resembling the indolent phenotypes driving metastatic latency described by Montagner et al. [59]. When further stratified by molecular subtype, Luminal A tumours showed a trend towards improved, but not significant, RFS in quiescent cases (*p* = 0.090), while Luminal B (*p* = 0.288), Her2+ (*p* = 0.629) and TNBC (*p* = 0.29) tumours showed no difference (Fig. S2k). PR+ tumours however, demonstrated a highly significant survival advantage for quiescent cases (*p* < 0.0001; Fig. 2j). These findings indicate that the G0 persister score does not simply recapitulate the molecular subtypes of breast cancer but rather reflects an independent clinically relevant feature, particularly in hormone receptor-positive tumours. However, we note that our power to capture significant differences is reduced in hormone-receptor negative cancers due to lower sample numbers.

### Genomic drivers of proliferation/G0 arrest capacity in single cells and large cancer cohorts

**Fig. 3:**
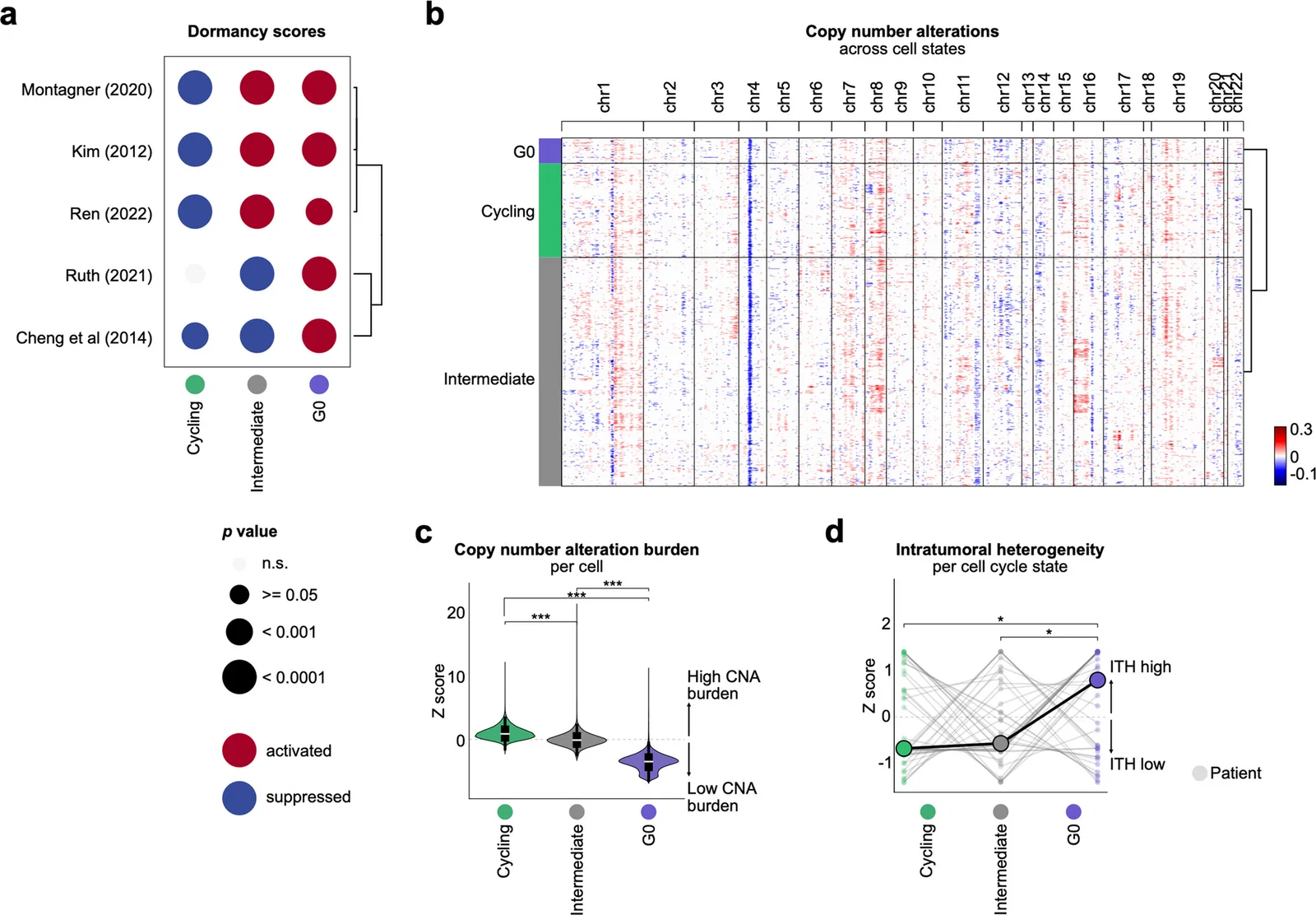
Genomic characterisation and trajectory analysis of cell cycle states. **a** Dormancy gene signatures across cell cycle states. **b** Heatmap of inferred copy number alterations across chromosomes for G0 persisters, cycling and intermediate cells. Red indicates amplifications; blue indicates deletions. **c** Violin plots showing CNA burden (z-score) per cell across cell cycle states. ***p < 0.001, Wilcoxon rank-sum test. **d** Bar plot of intratumoral heterogeneity (z-score) per cell cycle state, showing elevated heterogeneity in G0 persister cells. Each dot represents intratumoral heterogeneity score per patient. *p < 0.05, Wilcoxon rank sum test

Cancer cells often exit the cell cycle as an adaptive response to stress during tumour evolution. While the intermediate cells in our cohort likely represent a transient, short-lived G0 state, the G0 persisters may be trapped in a deeper and more actively sustained arrest, akin to dormant precursors associated with late recurrence. Indeed, the G0 persister cells activated dormancy programmes previously reported in breast cancer (Fig. 3a). To further confirm the nature of this state, we examined the genomic profiles of cycling, intermediate and G0 persister cells. G0 persisters displayed fewer CNA alterations (Fig. 3b-c), suggesting these cells may be “*stuck*” at an earlier tumour evolutionary stage before the acquisition of additional alterations. It is therefore plausible that these cells have been arrested for longer than a mere transient stress response. Differences in intratumoral CNA heterogeneity between cell cycle states were modest and showed substantial variability across patients, although G0 persisters tended to display higher heterogeneity overall (Fig. 3d). While the biological significance of this pattern remains unclear, it may suggest that G0 arrested populations retain greater clonal diversity than actively proliferating populations. Longitudinal studies will be required to test this hypothesis.

Gene-level CNA analysis revealed a limited and heterogeneous set of amplifications and deletions enriched in G0 persisters, including events affecting cell cycle regulators such as *RB1* and *CDKN1A* (p21) [78, 79] (Fig. S3a). However, these were not consistently recapitulated in bulk cohorts from TCGA and MET500 (Fig. S3b-c). These results suggest that no recurrent gene-level CNA events strongly define the G0 persister state.

Overall, differences in genomic features between cycling and G0 persister cells suggest that genomic context may contribute to variability at the extremes of cell cycle behaviour, consistent with the concept of “*heritable plasticity*” described by Schiffman et al. [80], although it is not sufficient to drive these states, which are likely shaped by non-genetic and microenvironmental factors.

### Master regulators of proliferation-arrest switches during breast cancer evolution

**Fig. 4:**
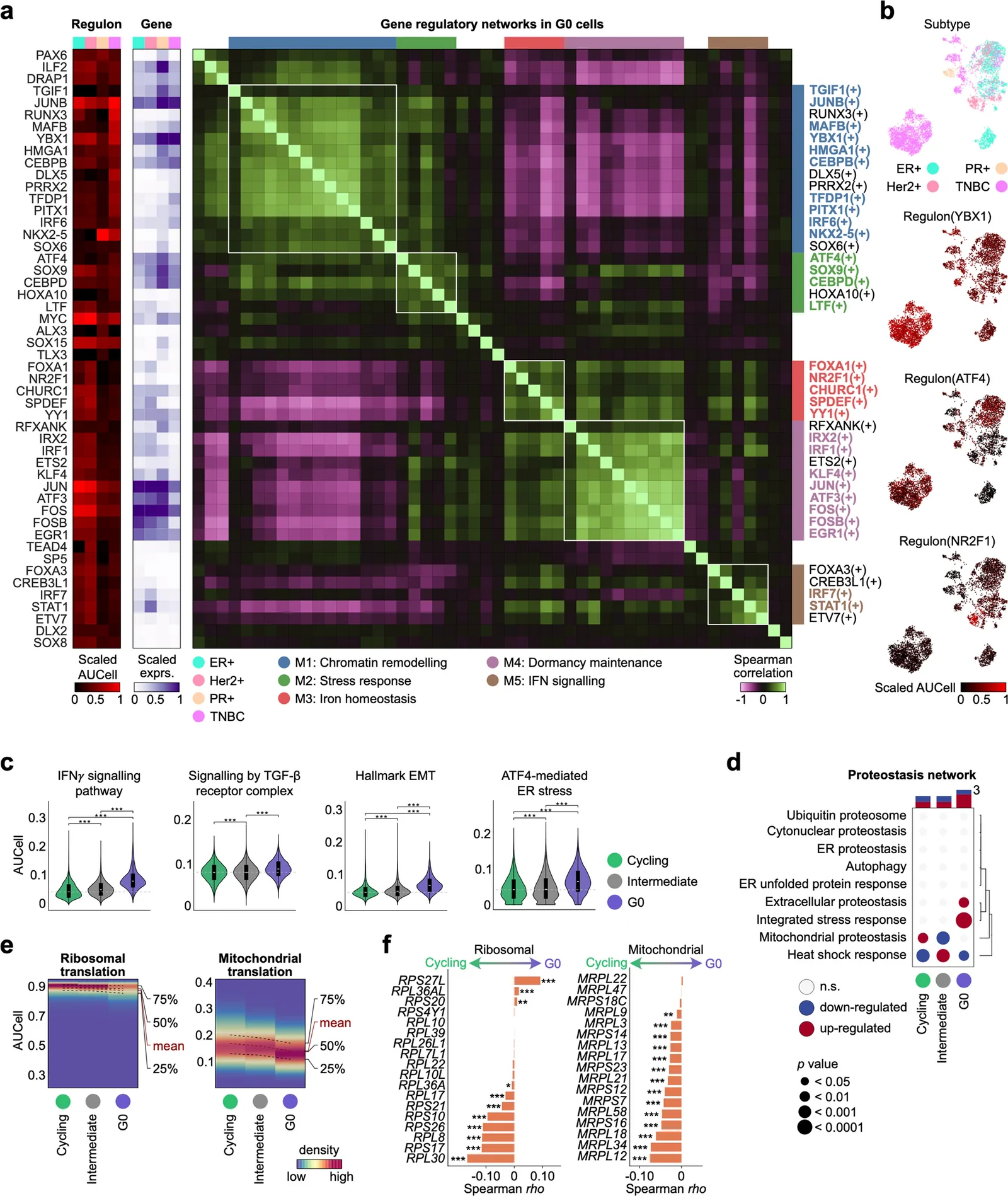
Gene regulatory networks and proteostasis remodelling in G0 persister cells. **a** Heatmap showing GRNs in G0 cells. The left heatmaps show AUCell enrichment scores and gene expression for each regulon across breast cancer subtypes, as inferred by SCENIC. The main heatmap displays Spearman correlations between regulon activities across molecular subtypes. Regulon modules are annotated by subtype specificity. **b** UMAP plots showing molecular subtype distribution and regulon activity scores for representative TFs. **c** Violin plots comparing pathway activity (AUCell scores) across cell cycle states for IFNγ signalling pathway, TGF-β receptor signalling, EMT and ATF4-mediated ER stress response. ***p < 0.001, Kruskal–Wallis test. **d** Dot plot of proteostasis network components showing differential regulation across cell cycle state. **e** Density plots showing distribution of ribosomal (left) and mitochondrial (right) translation enrichment across cell cycle states, with mean, 25^th^, 50^th^ and 75^th^ percentiles. **f** Correlation bar plots of individual ribosomal (left) and mitochondrial (right) gene expression with G0 score (***p < 0.001, **p < 0.01, *p < 0.05)

We explored master regulators of cell cycle-driven adaptation in breast cancer using SCENIC [64]. In G0 persister cells, we identified distinct transcription factor (TF) modules organised by their co-regulation patterns across molecular subtypes (Fig. 4a). These included stress-response regulons featuring ATF3, ATF4, KLF4, JUN, FOS and EGR1 [81], alongside dormancy-associated factors such as NR2F1 and FOXA1. Notably, NR2F1 showed prominent regulon activity specifically within G0 persisters, consistent with its established role in tumour dormancy [82], and mainly within the Her2 + subtype (Fig. 4b). G0 cells displayed elevated interferon gamma (IFNγ) and TGF-β receptor signalling [83], EMT transformation, increased ATF4 activity and integrated stress response (ISR) compared to proliferating populations (Fig. 4c). Module-based pathway enrichment analysis further revealed translation control and metabolic stress adaptation, mitochondrial metabolism and p53-linked bioenergetics, extracellular matrix (ECM) remodelling and adaptive immune activation in these cells (Fig. S5a; Table S3).

Cycling cells were governed by regulatory programmes driven by FOXM1, MYBL2, TFDP1 and E2F-associated factors (Fig. S4a-b). These transcription factors constitute a core regulatory circuitry that coordinates G2/M gene expression and mitotic progression [84, 85]. Gene expression modules in cycling cells included cell cycle governance programmes, as expected, but also growth factor and hormone-driven transcription, ECM remodelling, contractility, lipid metabolism and immune stress responses (Fig. S5b).

Intermediate cells activated gene regulatory networks shared by both cycling and G0 persister cells, including ATF4, ATF5, MYC and HDAC2 as core TFs (Fig. S4c, Fig. S5c). The co-activation of stress-responsive and proliferative programmes may represent a "*poised*" state that allows cells to rapidly commit to either fate in response to microenvironmental cues [86–88].

### Proteostasis pathways govern stress adaptation and G0 persistence in breast cancer

Proteostasis pathways are critical for maintaining protein folding, degradation and overall proteome integrity, enabling cells to adapt to stress and balance G0 arrest and proliferation [89]. Since our regulatory network analysis had highlighted proteostasis regulators like ATF3 and ATF4 (Fig. 4a, Fig. S5a), we further investigated whether proteostasis pathways may be differentially utilised by tumour cells depending on their state. G0 persister cells showed upregulation of the ISR, consistent with our observation of elevated ATF4 regulon activity in this population (Fig. 4a-c), alongside elevated extracellular proteostasis (Fig. 4d). Cycling cells upregulated mitochondrial proteostasis, while heat shock response was elevated in intermediate cells. While ribosomal transcription was similarly active in all three states, mitochondrial transcription showed marked reduction in G0 persisters (Fig. 4e-f), reflecting their lower energy demands [87, 90].

The preservation of cytoplasmic translation capacity despite reduced mitochondrial activity may represent an energy-conserving state that maintains the cellular machinery necessary for rapid reactivation [91], while the concurrent activation of stress response pathways could provide cytoprotection during prolonged quiescence. However, the activity of the translational machinery cannot be inferred from gene expression alone, and direct measurements of cytoplasmic and mitochondrial translation in G0 and cycling cells should be performed in the future to ascertain any effects on translation. To provide further support for this hypothesis, we examined translation initiation and elongation factor expression across cell cycle states (Fig. S6a-b), however, we did not observe a differentially altered response across cell states.

Overall, these analyses demonstrate the remarkable capacity of arrested cells to remodel their proteostasis network transcriptionally, potentially supporting a “*slow flow*” state of reduced but viable activity within the tumour, although further research is required to understand whether these changes are reflected at the level of protein synthesis and function.

### Cell cycle state-dependent rewiring of tumour cell-TME interactions in single cells

**Fig. 5:**
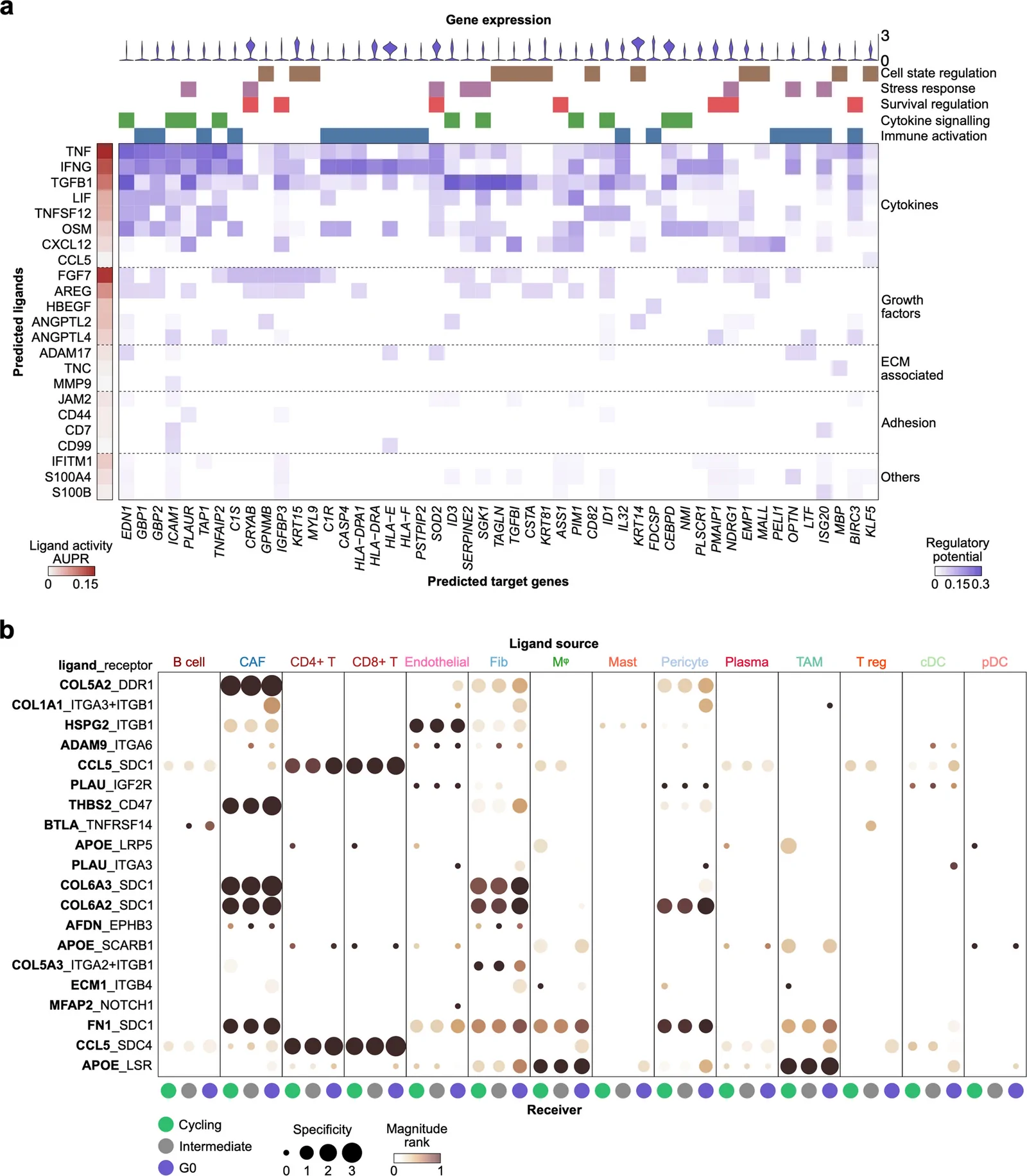
Tumour microenvironment communication with cell cycle states. **a** NicheNet ligand-target regulatory potential analysis for G0 persister cells. Rows highlight ligands on immune and stromal cells, alongside their activity depicted by a white-brown colour gradient. The ligands are grouped manually into biologically relevant categories, annotated to the right. Columns depict downstream targets in G0 cancer cells. Violin plots (top) display the expression levels of these target genes, followed by functional annotations of each gene. **b** Ligand-receptor interactions between G0 persisters, intermediate and cycling cancer cells, as inferred by CellPhoneDB. Ligands are shown on the left, receptors are shown on the right. Analyses were performed on data integrated across all breast cancer subtypes

Given that the tumour ecosystem influences cell cycle decisions [92], we next interrogated whether tumour ecological interactions are rewired when cells switch between proliferation and cell cycle arrest. First, using NicheNet v2 [61], we identified cytokines including TNF, IFNG, TGFB1 as top modulators of G0 persister cells expressed by immune and stromal cells in the TME (Fig. 5a, Fig. S7a), driving the expression of genes involved in immune activation, cytokine signalling, stress response, survival and cell state regulation [93, 94]. These ligands regulate target genes including *EDN1*, *GBP1*, *GBP2*, *ICAM1*, *PLAUR* and downstream effectors of cell cycle arrest [95, 96]. Growth factors such as FGF7 and AREG, alongside ECM-associated ligands (TNC, MMP9) and adhesion molecules (JAM2, CD44, CD99), also contributed to the G0 regulatory network (Fig. 5a). In cycling cells, cytokines AREG, GRN and IGF1 strongly correlated with genes involved in cytoskeletal and spindle organisation, checkpoint control, transcription and chromatin regulation, DNA replication, S-phase and mitosis (Fig. S7b-c; Table S4). ECM-associated ligands (*LAMA4*, *TIMP3*, *COL8A1*), cell surface adhesion molecules (*NECTIN2*, *CD44*) and proteases (*ADAM12*, *PSEN1*) also regulated cycling cell programmes (Fig. S7b-c), reflecting the increased requirement for matrix remodelling and cell–cell contact during active proliferation.

CellPhoneDB [62] analysis indicated that, compared to cycling cells, G0 persister cells show stronger interactions with stromal and immune cells (Fig. S7d). This communication asymmetry may reflect the "*social rewiring*" of dormant cells as a mechanism to evade immune surveillance and resist microenvironmental proliferative cues [12, 15]. Ligand-receptor analysis revealed that collagen-integrin signalling (COL5A2-DDR1, COL1A1-ITGA3 + ITGB1, COL6A3-SDC1, COL6A2-SDC1) from CAFs and fibroblasts was prominent across all cell states (Fig. 5b), suggesting that ECM engagement is a fundamental requirement regardless of proliferative status. APOE signalling from macrophages (APOE-LRP5, APOE-SCARB1, APOE-LSR) similarly engaged all cell cycle states. APOE has been implicated in lipid metabolism and immune modulation within the TME [97], potentially supporting both proliferative and survival programmes. CCL5 signalling with SDC1 and SDC4 originating from T cells also showed broad engagement. Notably, BTLA-TNFRSF14 signalling from B cells was specific to intermediate and G0 persister cells (Fig. 5b), potentially reflecting engagement of inhibitory immune signalling pathways in slower cycling cells [98, 99]. PLAU-ITGA3 interactions from endothelial and dendritic cells were specific to G0 persisters, potentially indicating priming for motility and invasion [100].

We also observed distinct communication patterns between tumour cells depending on their cell cycle state, with cells in the intermediate state sending the strongest signals to all other tumour cells (Fig. S7e). While most signalling patterns were captured across all cell cycle states, we did observe TNC-SDC4 interactions involved in ECM sensing [101] exclusively between intermediate and G0 persister cells, while EFNB2-RHBDL2 signalling between intermediate and cycling cells may facilitate ephrin-mediated cell positioning and vascularisation [102]. No unique interactions were observed amongst G0 cells, but cycling cells communicated through specific desmosomal (DSC3-DSG3) interactions, suggesting enhanced cell–cell adhesion as would be expected for proliferating niches [103].

### Spatial organisation of proliferation and G0 arrest phenotypes unveils extensive niche remodelling

**Fig. 6:**
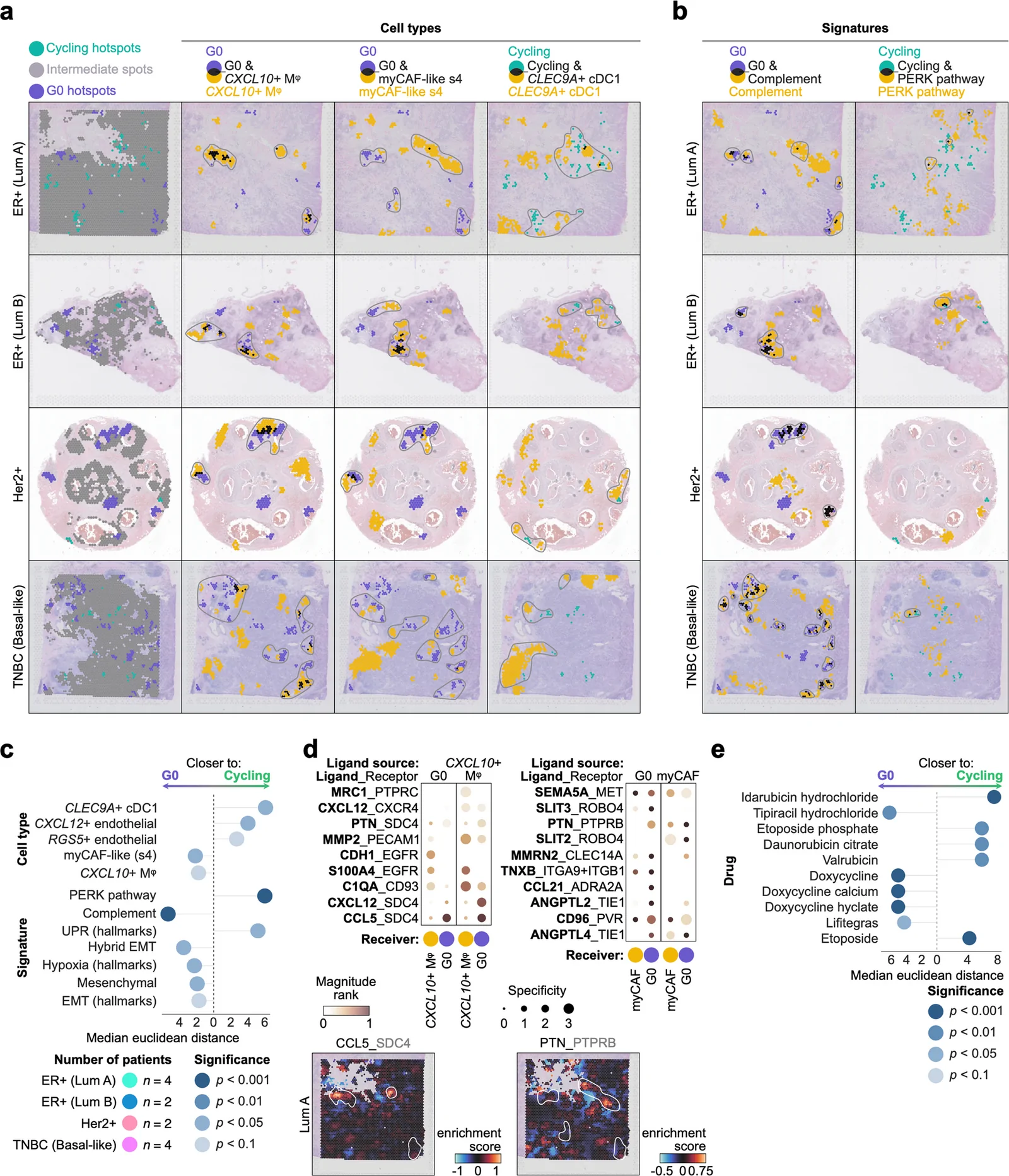
Spatial organisation of cell cycle states in breast cancer. **a** Spatial mapping of cell cycle state hotspots across breast cancer subtypes. First panel: Hotspots are colour-coded by cell cycle state: green (cycling) and purple (G0), while grey marks the remaining tumour spots. Subsequent panels: Co-localisation of G0 cells with *CXCL10*+ macrophage, myCAF-like fibroblasts and cycling cells with *CLEC9A*+ cDC1 cells. **b** Spatial mapping of pathway signatures. G0 hotspots with complement pathway activity; cycling hotspots with PERK pathway activity. **c** Median Euclidean distance analysis showing spatial proximity of cell types and pathway signatures to G0 versus cycling hotspots. Dot colour indicates statistical significance.** d** Top ligand-receptor interactions between G0 and *CXCL10*+ macrophage or myCAFs (dot plot) and spatial ligand-receptor interaction enrichment with white outlines indicating G0/*CXCL10*+ macrophage (bottom left) or G0/myCAF (bottom right) co-localisation in a Luminal A tumour. **e** Distances between drug-effective regions and either G0 or cycling hotspots

While single-cell data offer insights into cell cycle arrest regulation in cancer cells, the spatial emergence of these states within the tumour ecosystem remains unclear. To investigate this, we examined G0 arrest and proliferation phenotypes using 12 breast cancer 10x Visium slides across ER+ (Luminal A and Luminal B), Her2+ and TNBC (basal-like) subtypes (see Methods). Using our SpottedPy method [66], we mapped G0 and cycling tumour hotspots on these slides (Fig. 6a). The spatial patterning of these areas showed a good concordance with predicted Ki67 activity in the matched H&E stained images (Fig. S8a-b). While tissues were mainly composed of tumour cells with intermediate cycling capacity, the extreme proliferation or cell cycle arrest niches were dispersed throughout the tissue rather than co-located in a single region, suggestive of the tumour’s ability to fine-tune proliferation rates locally to adapt to varying stressors.

Across breast cancer subtypes, G0 hotspots were proximal to *CXCL10*+ macrophages and myofibroblastic cancer-associated fibroblasts (myCAFs) (Fig. 6a,c, Fig. S9a). In contrast, cycling regions were localised in the vicinity of *CLEC9A*+ conventional dendritic cells (cDCs) as well as *CXCL12*+ and *RGS5*+ endothelial populations, suggesting differential vascular engagement between proliferative and arrested states (Fig. 6a,c). Furthermore, G0 hotspots were enriched in complement pathway activity, hybrid EMT [54] and hypoxia, while cycling regions showed elevated PERK pathway signalling and unfolded protein response (UPR) activity (Fig. 6b-c).

When examining tumour niche factors by breast cancer subtype, we noticed that G0 hotspots appeared more immunogenic in ER+ breast cancers, where they were adjacent to CD4 and CD8 T cells alongside *EGR1*+ and *SIGLEC1*+ macrophages and myCAFs, whereas they appeared to be in immune absent areas in TNBC samples (Fig. S9b-c). This striking contrast may reflect subtype-specific adaptation, but we are unable to draw confident conclusions from this analysis due to the limited power of the reduced cohorts and the fact that TNBC slides presented fewer G0 hotspots. Complement pathway enrichment in G0 hotspots and PERK signalling linked with cycling regions were consistent across ER+ and TNBC cancers. Due to insufficient statistical power, hotspot analysis was not performed for Luminal B and Her2+ subtypes (*n* = 2 samples each).

Regardless of subtype-specificity, it is clear that the tumour niche appears to be extensively remodelled when cells switch from a proliferative to a G0 state, which may be enabled through secreted factors by the TME and/or by the G0 cancer cells themselves, as shown in our previous work [104]. To investigate this further, we performed spatial ligand-receptor analysis within the G0 hotspots. Enriched CDH1-EGFR, S100A4-EGFR and CCL5-SDC4 signalling was observed between *CXCL10*+ macrophages and G0 cells across breast cancer subtypes (Fig. 6d), indicative of programmes combining epithelial or hybrid EMT state maintenance (CDH1), EGFR-linked survival, motility (S100A4) and inflammatory communication (CCL5-SDC4). Additionally, myCAFs displayed enriched PTN-PTPRB signalling towards G0 cells (Fig. 6d), suggesting that myofibroblast-derived pleiotrophin may sustain quiescence by inhibiting PTPRB phosphatase activity in arrested tumour cells. Notably, PTN has been identified as a prometastatic niche factor in breast cancer [105], raising the possibility that the same myCAF-derived signal that maintains dormancy may also prime G0 cells for later metastatic dissemination.

To orthogonally validate these spatial patterns, we analysed Xenium spatial transcriptomics data at single-cell resolution (Fig. S10a). G0 persister scores mapped to tumour cells showed clear spatial segregation between G0 arrested and cycling populations, with the latter enriched in the more invasive regions, as expected (Fig. S10b-d). Furthermore, G0 cells were significantly closer to *CXCL10*+ macrophages and myCAFs when compared to cycling cells (Fig. S10e-h), confirming our previous results. Ligand-receptor analysis recapitulated several signals observed previously, including the S100A4-EGFR bidirectional signalling between G0 cancer cells and *CXCL10*+ macrophages. Consistent with our Visium findings, this analysis supports a model wherein G0 cells may evade cytotoxic immune surveillance through spatial positioning within protective macrophage and stromal niches.

### Segregation of G0 arrest and proliferative areas suggests differential sensitivities to drugs

The spatial segregation between G0 arrest and proliferative niches might inform therapy response. Using drug2cell [106], we identified molecular hotspots with distinct drug sensitivities. Spatial proximity analysis revealed that proliferative niches were significantly closer to areas predicted to respond to topoisomerase inhibitors and anthracyclines, including etoposide phosphate, daunorubicin citrate, valrubicin, teniposide and dexrazoxane, while G0 hotspots showed proximity to regions predicted to respond to tipiracil hydrochloride, doxycycline and its derivatives, and lifitegrast (Fig. 6e). Intermediate cells showed distinct sensitivity profiles, with enrichment for hormone-related compounds including danazol, estropipate, methyltestosterone, allylestrenol and diethylstilbestrol diphosphate.

Spatial visualisation of drug-niche associations across subtypes demonstrated that cycling regions co-localised with etoposide sensitivity hotspots, while G0 niches overlapped with tipiracil hydrochloride-responsive regions (Fig. S11). Etoposide, a topoisomerase II inhibitor, targets actively proliferating cells by inducing permanent DNA breaks that halt cell division in late S and G2 phases, and trigger cell death. Tipiracil, a thymidine phosphorylase inhibitor that prevents degradation of the cytotoxic agent trifluridine [107], and doxycycline, an antibiotic shown to inhibit cancer stem cells and potentiate chemotherapy effectiveness in breast cancer [108], may offer therapeutic potential for targeting quiescent tumour populations that are typically resistant to conventional chemotherapies targeting proliferating cells. Combined with our earlier findings that G0 niches display increased complement pathway activity (Fig. 6b-c), it is possible that combination strategies targeting both the metabolic vulnerabilities and immune evasion mechanisms within quiescent tumour niches could be therapeutically beneficial.

In summary, our analysis delineates distinct quiescent niches within primary tumours, rich in tumour-promoting signals associated with local stress adaptation. Surprisingly, these niches appear to be dispersed across the tumour, rather than clustering at the leading edge, hinting at proliferative and G0-hybrid EMT patterns that fluctuate throughout the tissue as a response to intrinsic and extrinsic stressors.

### A foundation model for G0 arrest and proliferation in single cells

To generalise G0 persister, intermediate and cycling state identification in unseen single-cell datasets, we employed a foundation model architecture similar to ones we have developed previously [72] to train a classifier of these states (Fig. S12a). The proposed single-cell Foundation Model (scFM), G0-FM, demonstrated robust performance in classifying these states, achieving an average AUROC score of 0.89 (Fig. S12b). Despite being trained solely for classification without prior temporal information, the model learned biologically informative representations that effectively distinguished G0 persister, intermediate and cycling cell states (Fig. S12c-d). These results highlight the model’s capacity to capture transcriptional features associated with distinct cellular phenotypes. To enable future analyses of G0, intermediate and proliferative phenotypes in single cell breast cancer data, we provide the G0-FM model at https://github.com/secrierlab/G0-FM [109].

## Discussion

Our study reveals the interplay of cell cycle states during breast cancer evolution, identifying proliferative and quiescent persister-like populations with distinct genetic and transcriptional adaptations shaped by the tumour ecosystem. We observed a subtype-dependent prevalence of these states: ER+ tumours harboured a greater proportion of G0 persisters and intermediate cells, while TNBC samples were rich in cycling cells (Fig. 2h), reflecting known differences in clinical outcomes. G0 persister cells displayed fewer CNAs and elevated intratumoral heterogeneity, alongside activation of dormancy programmes, suggesting they may be stably arrested at an earlier evolutionary stage rather than simply responding to transient stress. They were also found to display hybrid EMT characteristics, aligning with the *“go-or-grow*” model of cancer [27, 110–112], while exhibiting ISR signalling and reduced mitochondrial translation, allowing them to remain in a viable but metabolically idle state over prolonged periods. It has been shown that mitochondrial stress couples the activation of the ISR with reduced mitochondrial translation [87, 90]. This is highly consistent with our observations in G0 cells, suggesting that mitochondrial remodelling and metabolic adaptation may be what underlies the ISR-associated TF (i.e. ATF4, ATF3) activation. Since cytoplasmic ribosomal gene levels remained stable, translation may be reduced through ISR activation, allowing G0 cells to be “*primed*” for an increase in protein synthesis in case exit from G0 is required [91]. Validating this hypothesis through direct measurements of cytoplasmic and ribosomal translation will be essential in the future. These regulatory features are further reinforced by NR2F1 regulon activity, which is specifically enriched in G0 persister cells, consistent with its established role in maintaining tumour dormancy through SOX9- and RAR-β-driven quiescence programmes [82]. Together, the convergence of ISR activation, reduced mitochondrial translation and NR2F1-driven dormancy signalling supports the view that the G0 persister state is actively maintained rather than a passive consequence of low proliferative signalling.

Our study also highlights the role of the tumour ecological niche in sustaining cell cycle arrest. Spatial analyses revealed a G0 cancer ecosystem enriched in *CXCL10*+ macrophages and myCAFs, with complement pathway activity enriched near G0 hotspots. This niche is possibly sustained both by signals secreted by the *CXCL10*+ macrophages and myCAFs promoting an arrested, partially EMT-transformed state in tumour cells, and by secreted signals by the tumour cells themselves which may remodel the ECM and vasculature, co-opting tumour associated macrophages and myCAFs to evade immune recognition. The enrichment of *CXCL10*+ macrophages near G0 hotspots may reflect the presence of an interferon-responsive inflammatory microenvironment, consistent with the elevated interferon response and complement signalling observed in G0 cells. Recent work has implicated CXCL10 signalling in the maintenance of breast cancer dormancy [113], suggesting that inflammatory CXCL10-rich niches may contribute to long-term tumour persistence, although the causal role of macrophage-derived CXCL10 cytokine remains to be established. However, these associations were not uniformly observed across all patients, reflecting a level of interpatient heterogeneity that suggests that G0-associated ecosystems are likely context dependent rather than conserved across all breast tumours. Orthogonal validation using Xenium data further supports a model of immune evasion by G0 persisters through macrophage shielding, but experimental validation in co-culture systems will be essential in the future to confirm this. In contrast, cycling cells were spatially associated with *CLEC9A*+ cDC1 and PERK pathway activity, highlighting the need for therapies targeting distinct cancer-TME interactions to destabilise both types of niches.

While our findings align partly with Baldominos et al. [23], who noted a hypoxic quiescent niche containing immune-suppressive fibroblasts and exhausted T cells in TNBC tumours, we only observed T cells near G0 foci in ER+ tumours and not in TNBCs [23]. This is likely because Baldominos et al. [23] specifically selected T cell-resistant tumour cells, which are likely rarer quiescent cells situated within the cytotoxic and exhausted proliferative hotspots observed in our analyses. Thus, our analysis highlights a different G0 arrest phenotype within the evolving breast tumour that is likely not simply epigenetically driven as a reaction to T cell attack.

The spatial segregation between G0 arrest and proliferative niches may have therapeutic implications. Drug sensitivity analysis revealed an association between cycling regions and topoisomerase inhibitor and anthracycline response, while G0 hotspots localised in tipiracil and doxycycline-responsive regions. These findings suggest that combination strategies targeting both proliferating and quiescent populations may be necessary to prevent relapse from residual G0 cells.

Together, these analyses lay the groundwork for future studies, once several limitations are addressed. Our definition of G0 arrest relies on a tumour-specific signature validated against experimentally measured quiescent fractions, with thresholds calibrated against EdU incorporation proxies; however, these could be adjusted to capture gradual state changes, especially in spatial data. Since the dynamic emergence and extinction of cancer niches cannot be inferred from a single time point, future studies should employ longitudinally profiled samples from primary and matched metastatic tumours to gain insights into the stability of these niches and potential to form migratory precursors to metastasis. Secondly, CNA inference from scRNA-seq can be prone to error and susceptible to the reference employed. Future studies should combine DNA/RNA-sequencing with lineage tracing to draw more robust conclusions regarding the dynamics of cycling-G0 arrest decisions. Thirdly, the limited number of spatial transcriptomics samples (Luminal A, *n* = 4; Luminal B, *n* = 2; Her2+, *n* = 2; TNBC, *n* = 4) may not fully capture interpatient heterogeneity, and the lack of single-cell resolution of Visium spots complicates the inference of the origin of signals. Consequently, direct comparisons between spatially derived G0 enrichment and single-cell estimates of cycling or G0 cell proportions should be interpreted cautiously, particularly for subtypes represented by small sample numbers. Xenium validation at single-cell resolution partially addresses this limitation but was restricted to a single sample and a limited gene panel.

Although seminal studies such as that by Rosano et al. [22] noted that long-term dormancy is primarily driven by non-genetic adaptations to endocrine therapies, our analyses suggest that, at least in a pre-treatment setting, genomic alterations may predispose tumour cells to favour either proliferation or prolonged arrest [22]. Further studies employing methylation, chromatin conformation and metabolomics are needed to explore the non-genetic mechanisms contributing to the evolutionary role of G0 arrest in cancer.

Beyond its application to treatment-naïve primary tumours, our analysis pipeline could be extended to longitudinal and treatment-response settings. For instance, applying this framework to matched pre- and post-treatment biopsies would enable tracking of how the balance between G0 persister and cycling populations shifts in response to therapy, revealing whether G0-associated niches are remodelled by the treatment or whether they remain stable sanctuaries for drug-tolerant cells. Our G0-FM foundation model, trained to classify cell cycle states from single-cell transcriptomes, could further facilitate such studies by enabling rapid annotation of G0 states in newly generated datasets without the need for *de novo* signature calibration. Integrating this framework with emerging technologies such as spatial multi-omics and lineage tracing would provide a more complete picture of how proliferation-dormancy dynamics evolve across the disease trajectory and inform the design of combination strategies aimed at eliminating both proliferative and quiescent tumour compartments.

## Conclusions

Our study advances the understanding of breast cancer evolution by revealing critical ecological principles that govern cellular adaptation and niche specialisation. We identify a population of cancer cells within primary tumours that are transcriptionally similar to quiescent drug-tolerant persister cells whose state may be partially genetically constrained, and that reside in spatial niches protected by macrophage and myCAF-enriched microenvironments. These insights are particularly relevant for ER+ cancers, where targeting quiescent populations and disrupting dormancy-associated signalling networks, such as complement and cytokine-mediated pathways, may reduce late recurrence risk. Additionally, our spatial mapping of proliferation and G0 arrest hotspots highlights the need to tailor combination therapies to the unique microenvironmental context of each niche.

## Supplementary Information

Supplementary Material 1.

Supplementary Material 2.

Supplementary Material 3.

Supplementary Material 4.

Supplementary Material 5.

## Data Availability

The single-cell RNA sequencing datasets used in this study are publicly available through the Curated Cancer Cell Atlas of the Weizmann Institute of Science [74] (https://www.weizmann.ac.il/sites/3CA/breast), with raw files accessible via the Gene Expression Omnibus (GEO) under accession numbers GSE148673 [38] and GSE161529 [39], and through the ArrayExpress database of EMBL-EBI under accession number E-MTAB-8107 [37]. The tumour-specific G0 persister-like signature was derived from the single-cell RNA-seq dataset of Oren et al. [40] , available via GEO under accession number GSE150949 [40]. Xenium spatial transcriptomics data for breast cancer was obtained from [43], available through 10x Genomics (https://www.10xgenomics.com/datasets) [42]. For scoring Xenium slides with *CXCL10*+ macrophages and *LAG3*+ T cells, gene signatures were generated from Wu et al. [35]. Pre-processed and annotated single-cell Seurat objects are available on Zenodo [115] (https://doi.org/10.5281/zenodo.14001194). Gene sets for calculating G0 score are available at https://github.com/secrierlab/G0-breast-cancer-atlas [116] and in the Table S2. Processed spatial transcriptomics data is also publicly available on Zenodo [117] (https://doi.org/10.5281/zenodo.10371890). All scripts used in this study are publicly available at https://github.com/secrierlab/G0-breast-cancer-atlas [116]. The G0-FM model is available at https://github.com/secrierlab/G0-FM [109].

## Abbreviations

CAF: Cancer-associated fibroblast
cDC1: Conventional type 1 dendritic cell
CNA: Copy number alteration
DCIS: Ductal carcinoma *in situ*
DREAM: Dimerisation partner, Retinoblastoma-like, E2F and multi-vulval class B
ECM: Extracellular matrix
EdU: 5-Ethynyl-2′-deoxyuridine
EGFR: Epidermal growth factor receptor
EMT: Epithelial–mesenchymal transition
ER: Oestrogen receptor
FPKM: Fragments per kilobase of transcript per million mapped reads
G0-FM: G0 foundation model
GEO: Gene Expression Omnibus
GRN: Gene regulatory network
GSVA: Gene set variation analysis
HLA: Human leukocyte antigen
HMM: Hidden Markov model
ISR: Integrated stress response
ITH: Intratumoral heterogeneity
LoRA: Low-rank adaptation
LR: Ligand–receptor
METABRIC: Molecular Taxonomy of Breast Cancer International Consortium
MET500: Metastatic Cancer Project 500
MLP: Multilayer perceptron
myCAF: Myofibroblastic cancer-associated fibroblast
PEFT: Parameter-efficient fine-tuning
PERK: Protein kinase R-like endoplasmic reticulum kinase
PR: Progesterone receptor
SCENIC: Single-cell regulatory network inference and clustering
scFM: Single-cell foundation model
STARCH: Spatial transcriptomics-based analysis of regional copy number heterogeneity
TCGA: The Cancer Genome Atlas
TF: Transcription factor
TME: Tumour microenvironment
TNBC: Triple-negative breast cancer
UMAP: Uniform manifold approximation and projection
UPR: Unfolded protein response

## References

[1] Hanahan D. Hallmarks of cancer: new dimensions. Cancer Discov. 2022;12(1):31–46. 10.1158/2159-8290.CD-21-1059 35022204

[2] Ebinger S, Ozdemir EZ, Ziegenhain C, Tiedt S, Castro Alves C, Grunert M, et al. Characterization of rare, dormant, and therapy-resistant cells in acute lymphoblastic leukemia. Cancer Cell. 2016;30(6):849–62. 10.1016/j.ccell.2016.11.002 PMC5156313 27916615

[3] Francescangeli F, De Angelis ML, Rossi R, Cuccu A, Giuliani A, De Maria R, et al. Dormancy, stemness, and therapy resistance: interconnected players in cancer evolution. Cancer Metastasis Rev. 2023;42(1):197–215. 10.1007/s10555-023-10092-4 PMC10014678 36757577

[4] Agudo J, Aguirre-Ghiso JA, Bhatia M, Chodosh LA, Correia AL, Klein CA. Targeting cancer cell dormancy. Nat Rev Cancer. 2024;24(2):97–104. 10.1038/s41568-023-00642-x PMC11038906 38062251

[5] Phan TG, Croucher PI. The dormant cancer cell life cycle. Nat Rev Cancer. 2020;20(7):398–411. 10.1038/s41568-020-0263-0 32488200

[6] Weston WA, Barr AR. A cell cycle centric view of tumour dormancy. Br J Cancer. 2023;129(10):1535–45. 10.1038/s41416-023-02401-z PMC10645753 37608096

[7] Truskowski K, Amend SR, Pienta KJ. Dormant cancer cells: programmed quiescence, senescence, or both? Cancer Metastasis Rev. 2023;42(1):37–47. 10.1007/s10555-022-10073-z PMC10014758 36598661

[8] Pu Y, Li L, Peng H, Liu L, Heymann D, Robert C, et al. Drug-tolerant persister cells in cancer: the cutting edges and future directions. Nat Rev Clin Oncol. 2023;20(11):799–813. 10.1038/s41571-023-00815-5 37749382

[9] Recasens A, Munoz L. Targeting cancer cell dormancy. Trends Pharmacol Sci. 2019;40(2):128–41. 10.1016/j.tips.2018.12.004 30612715

[10] Aissa AF, Islam A, Ariss MM, Go CC, Rader AE, Conrardy RD, et al. Single-cell transcriptional changes associated with drug tolerance and response to combination therapies in cancer. Nat Commun. 2021;12(1):1628. 10.1038/s41467-021-21884-z PMC7955121 33712615

[11] Xue JY, Zhao Y, Aronowitz J, Mai TT, Vides A, Qeriqi B, et al. Rapid non-uniform adaptation to conformation-specific KRAS(G12C) inhibition. Nature. 2020;577(7790):421–5. 10.1038/s41586-019-1884-x PMC7308074 31915379

[12] Malladi S, Macalinao DG, Jin X, He L, Basnet H, Zou Y, et al. Metastatic Latency and Immune Evasion through Autocrine Inhibition of WNT. Cell. 2016;165(1):45–60. 10.1016/j.cell.2016.02.025 PMC4808520 27015306

[13] Ribas A. Adaptive immune resistance: how cancer protects from immune attack. Cancer Discov. 2015;5(9):915–9. 10.1158/2159-8290.CD-15-0563 PMC4560619 26272491

[14] Barkan D, El Touny LH, Michalowski AM, Smith JA, Chu I, Davis AS, et al. Metastatic growth from dormant cells induced by a col-I-enriched fibrotic environment. Cancer Res. 2010;70(14):5706–16. 10.1158/0008-5472.CAN-09-2356 PMC3436125 20570886

[15] Sosa MS, Bragado P, Aguirre-Ghiso JA. Mechanisms of disseminated cancer cell dormancy: an awakening field. Nat Rev Cancer. 2014;14(9):611–22. 10.1038/nrc3793 PMC4230700 25118602

[16] Ashraf HM, Moser J, Spencer SL. Senescence evasion in chemotherapy: a sweet spot for p21. Cell. 2019;178(2):267–9. 10.1016/j.cell.2019.06.025 PMC6805149 31299197

[17] Basile KJ, Aplin AE. Resistance to chemotherapy: short-term drug tolerance and stem cell-like subpopulations. Adv Pharmacol. 2012;65:315–34. 10.1016/B978-0-12-397927-8.00010-5 PMC6874398 22959030

[18] Harbeck N, Penault-Llorca F, Cortes J, Gnant M, Houssami N, Poortmans P, et al. Breast cancer. Nat Rev Dis Primers. 2019;5(1):66. 10.1038/s41572-019-0111-2 31548545

[19] Nolan E, Lindeman GJ, Visvader JE. Deciphering breast cancer: from biology to the clinic. Cell. 2023;186(8):1708–28. 10.1016/j.cell.2023.01.040 36931265

[20] Aouad P, Quinn HM, Berger A, Brisken C. Tumor dormancy: EMT beyond invasion and metastasis. Genesis. 2024;62(1):e23552. 10.1002/dvg.23552 37776086

[21] Aouad P, Zhang Y, De Martino F, Stibolt C, Ali S, Ambrosini G, et al. Epithelial-mesenchymal plasticity determines estrogen receptor positive breast cancer dormancy and epithelial reconversion drives recurrence. Nat Commun. 2022;13(1):4975. 10.1038/s41467-022-32523-6 PMC9411634 36008376

[22] Rosano D, Sofyali E, Dhiman H, Ghirardi C, Ivanoiu D, Heide T, et al. Long-term multimodal recording reveals epigenetic adaptation routes in dormant breast cancer cells. Cancer Discov. 2024;14(5):866–89. 10.1158/2159-8290.CD-23-1161 PMC11061610 38527495

[23] Baldominos P, Barbera-Mourelle A, Barreiro O, Huang Y, Wight A, Cho JW, et al. Quiescent cancer cells resist T cell attack by forming an immunosuppressive niche. Cell. 2022;185(10):1694-708 e19. 10.1016/j.cell.2022.03.033 PMC11332067 35447074

[24] Malla SB, Byrne RM, Lafarge MW, Corry SM, Fisher NC, Tsantoulis PK, et al. Pathway level subtyping identifies a slow-cycling biological phenotype associated with poor clinical outcomes in colorectal cancer. Nat Genet. 2024;56(3):458–72. 10.1038/s41588-024-01654-5 PMC10937375 38351382

[25] Qin X, Cardoso Rodriguez F, Sufi J, Vlckova P, Claus J, Tape CJ. An oncogenic phenoscape of colonic stem cell polarization. Cell. 2023;186(25):5554-68 e18. 10.1016/j.cell.2023.11.004 38065080

[26] Tape CJ. Plastic persisters: revival stem cells in colorectal cancer. Trends Cancer. 2024;10(3):185–95. 10.1016/j.trecan.2023.11.003 38071119

[27] Antonica F, Santomaso L, Pernici D, Petrucci L, Aiello G, Cutarelli A, et al. A slow-cycling/quiescent cells subpopulation is involved in glioma invasiveness. Nat Commun. 2022;13(1):4767. 10.1038/s41467-022-32448-0 PMC9378633 35970913

[28] Coller HA, Sang L, Roberts JM. A new description of cellular quiescence. PLoS Biol. 2006;4(3):e83. 10.1371/journal.pbio.0040083 PMC1393757 16509772

[29] Marescal O, Cheeseman IM. Cellular mechanisms and regulation of quiescence. Dev Cell. 2020;55(3):259–71. 10.1016/j.devcel.2020.09.029 PMC7665062 33171109

[30] Barr AR, Cooper S, Heldt FS, Butera F, Stoy H, Mansfeld J, et al. DNA damage during S-phase mediates the proliferation-quiescence decision in the subsequent G1 via p21 expression. Nat Commun. 2017;8:14728. 10.1038/ncomms14728 PMC5364389 28317845

[31] Heldt FS, Barr AR, Cooper S, Bakal C, Novak B. A comprehensive model for the proliferation-quiescence decision in response to endogenous DNA damage in human cells. Proc Natl Acad Sci U S A. 2018;115(10):2532–7. 10.1073/pnas.1715345115 PMC5877942 29463760

[32] Attieh Y, Vignjevic DM. The hallmarks of CAFs in cancer invasion. Eur J Cell Biol. 2016;95(11):493–502. 10.1016/j.ejcb.2016.07.004 27575401

[33] Rakina M, Larionova I, Kzhyshkowska J. Macrophage diversity in human cancers: New insight provided by single-cell resolution and spatial context. Heliyon. 2024;10(7):e28332. 10.1016/j.heliyon.2024.e28332 PMC10988020 38571605

[34] Sammut SJ, Crispin-Ortuzar M, Chin SF, Provenzano E, Bardwell HA, Ma W, et al. Multi-omic machine learning predictor of breast cancer therapy response. Nature. 2022;601(7894):623–9. 10.1038/s41586-021-04278-5 PMC8791834 34875674

[35] Wu SZ, Al-Eryani G, Roden DL, Junankar S, Harvey K, Andersson A, et al. A single-cell and spatially resolved atlas of human breast cancers. Nat Genet. 2021;53(9):1334–47. 10.1038/s41588-021-00911-1 PMC9044823 34493872

[36] Wiecek AJ, Cutty SJ, Kornai D, Parreno-Centeno M, Gourmet LE, Tagliazucchi GM, et al. Genomic hallmarks and therapeutic implications of G0 cell cycle arrest in cancer. Genome Biol. 2023;24(1):128. 10.1186/s13059-023-02963-4 PMC10204193 37221612

[37] Gao R, Bai S, Henderson YC, Lin Y, Schalck A, Yan Y, et al. Delineating copy number and clonal substructure in human tumors from single-cell transcriptomes. Nat Biotechnol. 2021;39(5):599–608. 10.1038/s41587-020-00795-2 PMC8122019 33462507

[38] Pal B, Chen Y, Vaillant F, Capaldo BD, Joyce R, Song X, et al. A single-cell RNA expression atlas of normal, preneoplastic and tumorigenic states in the human breast. EMBO J. 2021;40(11):e107333. 10.15252/embj.2020107333 PMC8167363 33950524

[39] Qian J, Olbrecht S, Boeckx B, Vos H, Laoui D, Etlioglu E, et al. A pan-cancer blueprint of the heterogeneous tumor microenvironment revealed by single-cell profiling. Cell Res. 2020;30(9):745–62. 10.1038/s41422-020-0355-0 PMC7608385 32561858

[40] Oren Y, Tsabar M, Cuoco MS, Amir-Zilberstein L, Cabanos HF, Hutter JC, et al. Cycling cancer persister cells arise from lineages with distinct programs. Nature. 2021;596(7873):576–82. 10.1038/s41586-021-03796-6 PMC9209846 34381210

[41] Barkley D, Moncada R, Pour M, Liberman DA, Dryg I, Werba G, et al. Cancer cell states recur across tumor types and form specific interactions with the tumor microenvironment. Nat Genet. 2022;54(8):1192–201. 10.1038/s41588-022-01141-9 PMC9886402 35931863

[42] 10X Genomics I. 10X Genomics, Inc.; Available from: https://www.10xgenomics.com/datasets.

[43] Janesick A, Shelansky R, Gottscho AD, Wagner F, Williams SR, Rouault M, et al. High resolution mapping of the tumor microenvironment using integrated single-cell, spatial and in situ analysis. Nat Commun. 2023;14(1):8353. 10.1038/s41467-023-43458-x PMC10730913 38114474

[44] Robinson DR, Wu YM, Lonigro RJ, Vats P, Cobain E, Everett J, et al. Integrative clinical genomics of metastatic cancer. Nature. 2017;548(7667):297–303. 10.1038/nature23306 PMC5995337 28783718

[45] Hao Y, Hao S, Andersen-Nissen E, Mauck WM 3rd, Zheng S, Butler A, et al. Integrated analysis of multimodal single-cell data. Cell. 2021;184(13):3573-87 e29. 10.1016/j.cell.2021.04.048 PMC8238499 34062119

[46] Franzen O, Gan LM, Bjorkegren JLM. PanglaoDB: a web server for exploration of mouse and human single-cell RNA sequencing data. Database (Oxford). 2019;2019:baz046. 10.1093/database/baz046 PMC6450036 30951143

[47] Ianevski A, Giri AK, Aittokallio T. Fully-automated and ultra-fast cell-type identification using specific marker combinations from single-cell transcriptomic data. Nat Commun. 2022;13(1):1246. 10.1038/s41467-022-28803-w PMC8913782 35273156

[48] Li M, Zhang X, Ang KS, Ling J, Sethi R, Lee NYS, et al. DISCO: a database of deeply integrated human single-cell omics data. Nucleic Acids Res. 2022;50(D1):D596–602. 10.1093/nar/gkab1020 PMC8728243 34791375

[49] McDavid A, Finak G, Chattopadyay PK, Dominguez M, Lamoreaux L, Ma SS, et al. Data exploration, quality control and testing in single-cell qPCR-based gene expression experiments. Bioinformatics. 2013;29(4):461–7. 10.1093/bioinformatics/bts714 PMC3570210 23267174

[50] Tickle T, Tirosh I, Georgescu C, Brown M, Haas B. inferCNV of the Trinity CTAT Project Klarman Cell Observatory, Broad Institute of MIT and Harvard. 2019. Available from: https://github.com/broadinstitute/inferCNV.

[51] Min M, Spencer SL. Spontaneously slow-cycling subpopulations of human cells originate from activation of stress-response pathways. PLoS Biol. 2019;17(3):e3000178. 10.1371/journal.pbio.3000178 PMC6433297 30865623

[52] Zerjatke T, Gak IA, Kirova D, Fuhrmann M, Daniel K, Gonciarz M, et al. Quantitative cell cycle analysis based on an endogenous all-in-one reporter for cell tracking and classification. Cell Rep. 2017;19(9):1953–66. 10.1016/j.celrep.2017.05.022 PMC5464964 28564611

[53] Colaprico A, Silva TC, Olsen C, Garofano L, Cava C, Garolini D, et al. TCGAbiolinks: an R/Bioconductor package for integrative analysis of TCGA data. Nucleic Acids Res. 2016;44(8):e71. 10.1093/nar/gkv1507 PMC4856967 26704973

[54] Malagoli Tagliazucchi G, Wiecek AJ, Withnell E, Secrier M. Genomic and microenvironmental heterogeneity shaping epithelial-to-mesenchymal trajectories in cancer. Nat Commun. 2023;14(1):789. 10.1038/s41467-023-36439-7 PMC9922305 36774358

[55] Ren Q, Khoo WH, Corr AP, Phan TG, Croucher PI, Stewart SA. Gene expression predicts dormant metastatic breast cancer cell phenotype. Breast Cancer Res. 2022;24(1):10. 10.1186/s13058-022-01503-5 PMC8800302 35093137

[56] Ruth JR, Pant DK, Pan TC, Seidel HE, Baksh SC, Keister BA, et al. Cellular dormancy in minimal residual disease following targeted therapy. Breast Cancer Res. 2021;23(1):63. 10.1186/s13058-021-01416-9 PMC8178846 34088357

[57] Cheng Q, Chang JT, Gwin WR, Zhu J, Ambs S, Geradts J, et al. A signature of epithelial-mesenchymal plasticity and stromal activation in primary tumor modulates late recurrence in breast cancer independent of disease subtype. Breast Cancer Res. 2014;16(4):407. 10.1186/s13058-014-0407-9 PMC4187325 25060555

[58] Kim RS, Avivar-Valderas A, Estrada Y, Bragado P, Sosa MS, Aguirre-Ghiso JA, et al. Dormancy signatures and metastasis in estrogen receptor positive and negative breast cancer. PLoS ONE. 2012;7(4):e35569. 10.1371/journal.pone.0035569 PMC3329481 22530051

[59] Montagner M, Bhome R, Hooper S, Chakravarty P, Qin X, Sufi J, et al. Crosstalk with lung epithelial cells regulates Sfrp2-mediated latency in breast cancer dissemination. Nat Cell Biol. 2020;22(3):289–96. 10.1038/s41556-020-0474-3 PMC7610690 32094692

[60] Celik C, Secrier M. EnrichMap: Spatially informed enrichment analysis for functional interpretation of spatial transcriptomics. bioRxiv. 2025:2025.05.30.656960.

[61] Browaeys R, Saelens W, Saeys Y. NicheNet: modeling intercellular communication by linking ligands to target genes. Nat Methods. 2020;17(2):159–62. 10.1038/s41592-019-0667-5 31819264

[62] Troulé K, Petryszak R, Prete M, Cranley J, Harasty A, Tuong ZK, et al. CellPhoneDB v5: inferring cell-cell communication from single-cell multiomics data. arXiv:231104567. 2023. 10.1038/s41596-024-01137-1 40133495

[63] Dimitrov D, Schafer PSL, Farr E, Rodriguez-Mier P, Lobentanzer S, Badia IMP, et al. LIANA+ provides an all-in-one framework for cell-cell communication inference. Nat Cell Biol. 2024;26(9):1613–22. 10.1038/s41556-024-01469-w PMC11392821 39223377

[64] Aibar S, Gonzalez-Blas CB, Moerman T, Huynh-Thu VA, Imrichova H, Hulselmans G, et al. SCENIC: single-cell regulatory network inference and clustering. Nat Methods. 2017;14(11):1083–6. 10.1038/nmeth.4463 PMC5937676 28991892

[65] DeTomaso D, Yosef N. Hotspot identifies informative gene modules across modalities of single-cell genomics. Cell Syst. 2021;12(5):446-56 e9. 10.1016/j.cels.2021.04.005 33951459

[66] Withnell E, Secrier M. SpottedPy quantifies relationships between spatial transcriptomic hotspots and uncovers environmental cues of epithelial-mesenchymal plasticity in breast cancer. Genome Biol. 2024;25(1):289. 10.1186/s13059-024-03428-y PMC11552145 39529126

[67] Kleshchevnikov V, Shmatko A, Dann E, Aivazidis A, King HW, Li T, et al. Cell 2location maps fine-grained cell types in spatial transcriptomics. Nat Biotechnol. 2022;40(5):661–71. 10.1038/s41587-021-01139-4 35027729

[68] Elyanow R, Zeira R, Land M, Raphael BJ. STARCH: copy number and clone inference from spatial transcriptomics data. Phys Biol. 2021;18(3):035001. 10.1088/1478-3975/abbe99 PMC9876615 33022659

[69] Navab R, Strumpf D, To C, Pasko E, Kim KS, Park CJ, et al. Integrin alpha11beta1 regulates cancer stromal stiffness and promotes tumorigenicity and metastasis in non-small cell lung cancer. Oncogene. 2016;35(15):1899–908. 10.1038/onc.2015.254 PMC4833874 26148229

[70] Liu Y, Li X, Zheng A, Zhu X, Liu S, Hu M, et al. Predict Ki-67 positive cells in H&E-stained images using deep learning independently from IHC-stained images. Front Mol Biosci. 2020;7:183. 10.3389/fmolb.2020.00183 PMC7438787 32903653

[71] Yang F, Wang WC, Wang F, Fang Y, Tang DY, Huang JZ, et al. scBERT as a large-scale pretrained deep language model for cell type annotation of single-cell RNA-seq data. Nat Mach Intell. 2022;4(10):852.

[72] Pan S, Withnell E, Secrier M. Classifying epithelial-mesenchymal transition states in single cell cancer data using large language models. bioRxiv. 2024:2024.08.16.608311.

[73] Hu EJ, Shen Y, Wallis P, Zeyuan AZ, Li Y, Wang S, et al. LoRA: Low-Rank Adaptation of Large Language Models. arxiv preprint. 2021.

[74] Gavish A, Tyler M, Greenwald AC, Hoefflin R, Simkin D, Tschernichovsky R, et al. Hallmarks of transcriptional intratumour heterogeneity across a thousand tumours. Nature. 2023;618(7965):598–606. 10.1038/s41586-023-06130-4 37258682

[75] Cutty SJ, Hughes FA, Ortega-Prieto P, Desai S, Thomas P, Fets LV, et al. Pro-survival roles for p21(Cip1/Waf1) in non-small cell lung cancer. Br J Cancer. 2025;132(5):421–37. 10.1038/s41416-024-02928-9 PMC11876327 39706988

[76] Clarke ZA, Bader GD. MALAT1 expression indicates cell quality in single-cell RNA sequencing data. bioRxiv. 2024:2024.07.14.603469.

[77] O‘Connor SA, Garcia L, Hoover R, Patel AP, Bartelle BB, Hugnot J-P, et al. Classifying cell cycle states and a quiescent-like G0 state using single-cell transcriptomics. bioRxiv. 2025:2024.04.16.589816.

[78] Santoro A, Vlachou T, Luzi L, Melloni G, Mazzarella L, D’Elia E, et al. p53 loss in breast cancer leads to Myc activation, increased cell plasticity, and expression of a mitotic signature with prognostic value. Cell Rep. 2019;26(3):624-38 e8. 10.1016/j.celrep.2018.12.071 PMC6334229 30650356

[79] Uribe ML, Marrocco I, Yarden Y. EGFR in cancer: signaling mechanisms, drugs, and acquired resistance. Cancers (Basel). 2021;13(11):2748. 10.3390/cancers13112748 PMC8197917 34206026

[80] Schiffman JS, D’Avino AR, Prieto T, Pang Y, Fan Y, Rajagopalan S, et al. Defining heritability, plasticity, and transition dynamics of cellular phenotypes in somatic evolution. Nat Genet. 2024;56(10):2174–84. 10.1038/s41588-024-01920-6 PMC11527590 39317739

[81] Fan P, Jordan VC. PERK, beyond an unfolded protein response sensor in estrogen-induced apoptosis in endocrine-resistant breast cancer. Mol Cancer Res. 2022;20(2):193–201. 10.1158/1541-7786.MCR-21-0702 PMC8816868 34728551

[82] Sosa MS, Parikh F, Maia AG, Estrada Y, Bosch A, Bragado P, et al. NR2F1 controls tumour cell dormancy via SOX9- and RARbeta-driven quiescence programmes. Nat Commun. 2015;6:6170. 10.1038/ncomms7170 PMC4313575 25636082

[83] Lan Q, Peyvandi S, Duffey N, Huang YT, Barras D, Held W, et al. Type I interferon/IRF7 axis instigates chemotherapy-induced immunological dormancy in breast cancer. Oncogene. 2019;38(15):2814–29. 10.1038/s41388-018-0624-2 PMC6477891 30546090

[84] Fischer M, Schade AE, Branigan TB, Muller GA, DeCaprio JA. Coordinating gene expression during the cell cycle. Trends Biochem Sci. 2022;47(12):1009–22. 10.1016/j.tibs.2022.06.007 35835684

[85] Sadasivam S, DeCaprio JA. The DREAM complex: master coordinator of cell cycle-dependent gene expression. Nat Rev Cancer. 2013;13(8):585–95. 10.1038/nrc3556 PMC3986830 23842645

[86] Pakos-Zebrucka K, Koryga I, Mnich K, Ljujic M, Samali A, Gorman AM. The integrated stress response. EMBO Rep. 2016;17(10):1374–95. 10.15252/embr.201642195 PMC5048378 27629041

[87] Quiros PM, Prado MA, Zamboni N, D’Amico D, Williams RW, Finley D, et al. Multi-omics analysis identifies ATF4 as a key regulator of the mitochondrial stress response in mammals. J Cell Biol. 2017;216(7):2027–45. 10.1083/jcb.201702058 PMC5496626 28566324

[88] Wortel IMN, van der Meer LT, Kilberg MS, van Leeuwen FN. Surviving Stress: Modulation of ATF4-Mediated Stress Responses in Normal and Malignant Cells. Trends Endocrinol Metab. 2017;28(11):794–806. 10.1016/j.tem.2017.07.003 PMC5951684 28797581

[89] Hipp MS, Kasturi P, Hartl FU. The proteostasis network and its decline in ageing. Nat Rev Mol Cell Biol. 2019;20(7):421–35. 10.1038/s41580-019-0101-y 30733602

[90] Mick E, Titov DV, Skinner OS, Sharma R, Jourdain AA, Mootha VK. Distinct mitochondrial defects trigger the integrated stress response depending on the metabolic state of the cell. Elife. 2020;9:e49178. 10.7554/eLife.49178 PMC7255802 32463360

[91] Smith PR, Pandit SC, Loerch S, Campbell ZT. The space between notes: emerging roles for translationally silent ribosomes. Trends Biochem Sci. 2022;47(6):477–91. 10.1016/j.tibs.2022.02.003 PMC9106873 35246374

[92] Tomasin R, Bruni-Cardoso A. The role of cellular quiescence in cancer - beyond a quiet passenger. J Cell Sci. 2022;135(15):jcs.259676. 10.1242/jcs.259676 35929545

[93] Bui JD, Schreiber RD. Cancer immunosurveillance, immunoediting and inflammation: independent or interdependent processes? Curr Opin Immunol. 2007;19(2):203–8. 10.1016/j.coi.2007.02.001 17292599

[94] Prunier C, Baker D, Ten Dijke P, Ritsma L. TGF-beta family signaling pathways in cellular dormancy. Trends Cancer. 2019;5(1):66–78. 10.1016/j.trecan.2018.10.010 30616757

[95] Derynck R, Turley SJ, Akhurst RJ. TGFbeta biology in cancer progression and immunotherapy. Nat Rev Clin Oncol. 2021;18(1):9–34. 10.1038/s41571-020-0403-1 PMC9721352 32710082

[96] Richardson L, Wilcockson SG, Guglielmi L, Hill CS. Context-dependent TGFbeta family signalling in cell fate regulation. Nat Rev Mol Cell Biol. 2023;24(12):876–94. 10.1038/s41580-023-00638-3 37596501

[97] Miao G, Zhuo D, Han X, Yao W, Liu C, Liu H, et al. From degenerative disease to malignant tumors: Insight to the function of ApoE. Biomed Pharmacother. 2023;158:114127. 10.1016/j.biopha.2022.114127 36516696

[98] Watanabe N, Gavrieli M, Sedy JR, Yang J, Fallarino F, Loftin SK, et al. BTLA is a lymphocyte inhibitory receptor with similarities to CTLA-4 and PD-1. Nat Immunol. 2003;4(7):670–9. 10.1038/ni944 12796776

[99] Ghoshdastider U, Rohatgi N, Mojtabavi Naeini M, Baruah P, Revkov E, Guo YA, et al. Pan-cancer analysis of ligand-receptor cross-talk in the tumor microenvironment. Cancer Res. 2021;81(7):1802–12. 10.1158/0008-5472.CAN-20-2352 33547160

[100] Zhang F, Tom CC, Kugler MC, Ching TT, Kreidberg JA, Wei Y, et al. Distinct ligand binding sites in integrin alpha3beta1 regulate matrix adhesion and cell-cell contact. J Cell Biol. 2003;163(1):177–88. 10.1083/jcb.200304065 PMC2173444 14557254

[101] Guan Z, Sun Y, Mu L, Jiang Y, Fan J. Tenascin-C promotes bladder cancer progression and its action depends on syndecan-4 and involves NF-kappaB signaling activation. BMC Cancer. 2022;22(1):240. 10.1186/s12885-022-09285-x PMC8896393 35246056

[102] Bochenek ML, Dickinson S, Astin JW, Adams RH, Nobes CD. Ephrin-B2 regulates endothelial cell morphology and motility independently of Eph-receptor binding. J Cell Sci. 2010;123(Pt 8):1235–46. 10.1242/jcs.061903 PMC2848112 20233847

[103] Dusek RL, Attardi LD. Desmosomes: new perpetrators in tumour suppression. Nat Rev Cancer. 2011;11(5):317–23. 10.1038/nrc3051 PMC3799918 21508970

[104] Weston WA, Holt JA, Wiecek AJ, Pilling J, Schiavone LH, Smith DM, et al. An image-based screen for secreted proteins involved in breast cancer G0 cell cycle arrest. Sci Data. 2024;11(1):868. 10.1038/s41597-024-03697-z PMC11316812 39127790

[105] Ganguly D, Schmidt MO, Coleman M, Ngo TC, Sorrelle N, Dominguez ATA, et al. Pleiotrophin drives a prometastatic immune niche in breast cancer. J Exp Med. 2023;220(5):e20220610. 10.1084/jem.20220610 PMC9998964 36828390

[106] Kanemaru K, Cranley J, Muraro D, Miranda AMA, Ho SY, Wilbrey-Clark A, et al. Spatially resolved multiomics of human cardiac niches. Nature. 2023;619(7971):801–10. 10.1038/s41586-023-06311-1 PMC10371870 37438528

[107] Prager GW, Taieb J, Fakih M, Ciardiello F, Van Cutsem E, Elez E, et al. Trifluridine-tipiracil and bevacizumab in refractory metastatic colorectal cancer. N Engl J Med. 2023;388(18):1657–67. 10.1056/NEJMoa2214963 37133585

[108] Zhang L, Xu L, Zhang F, Vlashi E. Doxycycline inhibits the cancer stem cell phenotype and epithelial-to-mesenchymal transition in breast cancer. Cell Cycle. 2017;16(8):737–45. 10.1080/15384101.2016.1241929 PMC5405729 27753527

[109] Pan S. G0-FM. GitHub. 2025. https://github.com/secrierlab/G0-FM/.

[110] Akhmetkaliyev A, Alibrahim N, Shafiee D, Tulchinsky E. EMT/MET plasticity in cancer and Go-or-Grow decisions in quiescence: the two sides of the same coin? Mol Cancer. 2023;22(1):90. 10.1186/s12943-023-01793-z PMC10230810 37259089

[111] Basu S, Dong Y, Kumar R, Jeter C, Tang DG. Slow-cycling (dormant) cancer cells in therapy resistance, cancer relapse and metastasis. Semin Cancer Biol. 2022;78:90–103. 10.1016/j.semcancer.2021.04.021 PMC8576068 33979674

[112] Landau DA, Carter SL, Stojanov P, McKenna A, Stevenson K, Lawrence MS, et al. Evolution and impact of subclonal mutations in chronic lymphocytic leukemia. Cell. 2013;152(4):714–26. 10.1016/j.cell.2013.01.019 PMC3575604 23415222

[113] Yilmaz A, Haerri L, Granda ME, Coquoz O, Lan Q, Ruegg C. The CXCL10/CXCR3 axis is essential for sustaining immunological dormancy in triple-negative breast cancer. NPJ Breast Cancer. 2026;12(1):36. 10.1038/s41523-026-00903-6 PMC12960658 41617729

[114] Malcolm JR, Stenning J, Pope J, Łukaszonek J, Rose SF, Smith TE, et al. An ERα-Dependent Hypoxia Response Defines EMT-Adjacent Tumour Regions and Suppresses the Pro-survival Effects of Amiloride in Estrogen Receptor-Positive Breast Cancer. bioRxiv. 2025. 10.1101/2025.10.03.680339.

[115] Celik C, Secrier M. Single-cell G0 breast cancer atlas. Zenodo. 2026. 10.5281/zenodo.21037473.

[116] Celik C. G0 breast cancer atlas. GitHub. 2026. https://github.com/secrierlab/G0-breast-cancer-atlas.

[117] Withnell E. Sample SpottedPy dataset. Zenodo. 2023. 10.5281/zenodo.10371890.

