# Supplementary material for: Spatial ecology of breast cancer reveals co-evolution of proliferative and dormant niches
Source: Genome Med. 2026 Jul 9;18:143. doi: 10.1186/s13073-026-01711-0 (PMC13632142; doi:10.1186/s13073-026-01711-0)
Supplement: Supplementary file 1 [file 13073_2026_1711_MOESM1_ESM.pdf]

SUPPLEMENTARY MATERIAL

Spatial ecology of breast cancer reveals co-evolution of proliferative and dormant niches

Cenk Celik[1], William A. Weston[2], Thais de Moraes-Lacerda[2,3], Eloise Withnell[1], Shi Pan[1], Tooki Chu[1], John Labbadia[4], Alexis R. Barr[2,5*], Maria Secrier[1,*]

[1]UCL Genetics Institute, Department of Genetics, Evolution and Environment, University College London, Gower Street, London, WC1E 6BT, UK

[2]Cell Cycle Control Team, MRC Laboratory of Medical Sciences, London, W12 0HS, UK

[3]NanoCell Interactions Lab, Departamento de Bioquímica e Biologia Tecidual, Instituto de Biologia, Universidade Estadual de Campinas (UNICAMP), Campinas, SP, Brazil

[4]Institute of Healthy Ageing, Department of Genetics, Evolution and Environment, University College London, Gower Street, London, WC1E 6BT, UK

[5]Institute of Clinical Sciences, Faculty of Medicine, Imperial College London, London, UK

*Corresponding authors: m.secrier@ucl.ac.uk and a.barr@lms.mrc.ac.uk

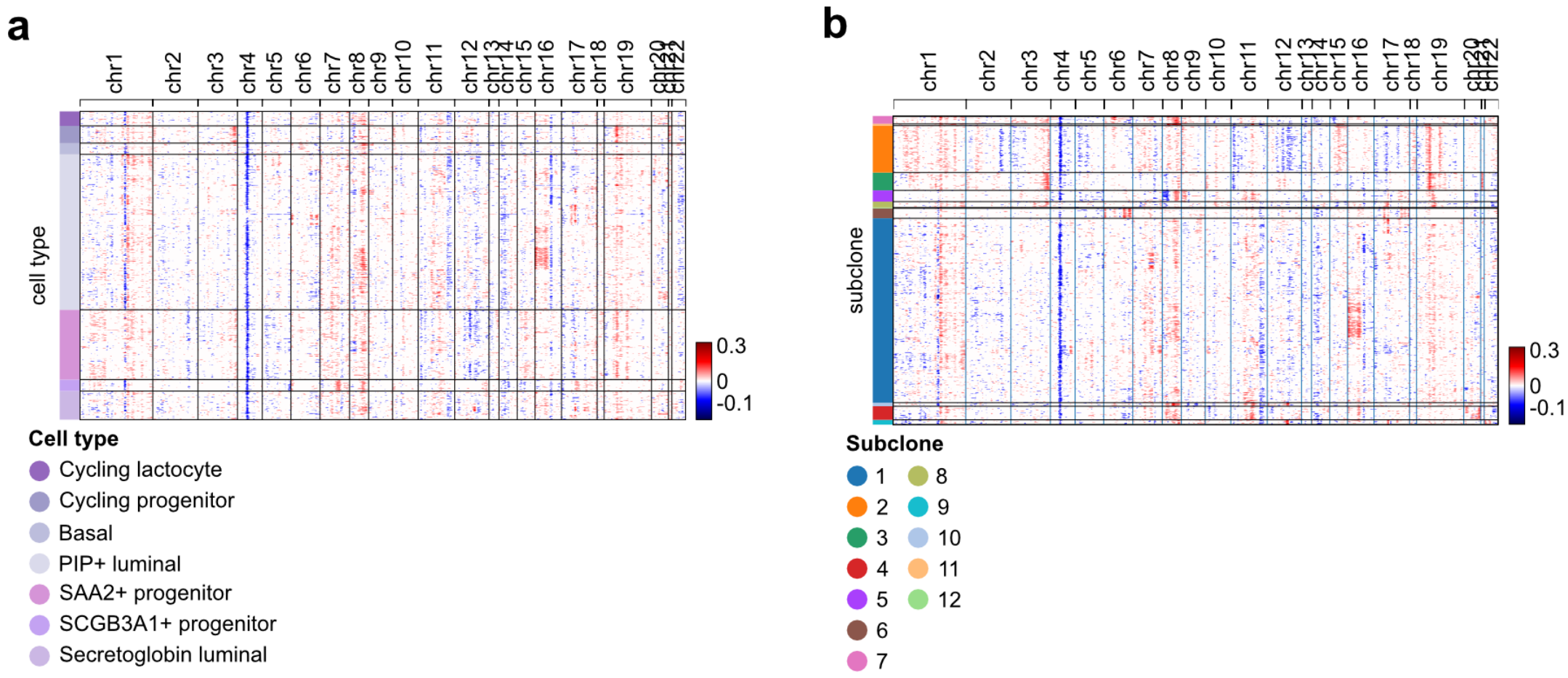

Fig. S1. Inferred copy number alterations in tumour cells.

a-b Inferred copy number alterations in epithelial cells grouped by cell type **(a)** and by subclone **(b)**.

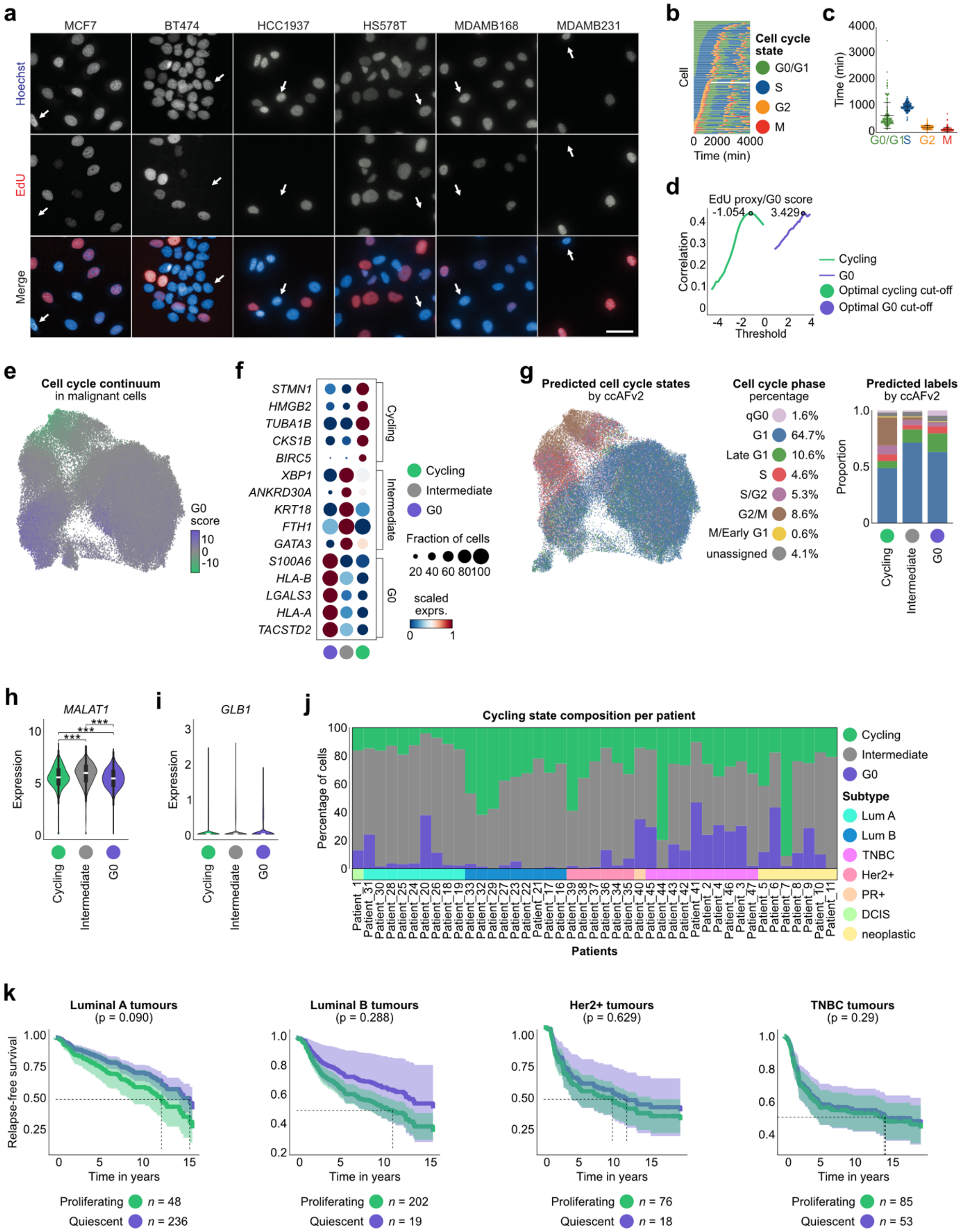

Fig. S2. Cell cycle state characterisation. a Images show breast cancer cell lines labelled with EdU for 24 h before fixing and imaging. White arrows point to examples of EdU negative (quiescent) cells. Hoechst is in blue and EdU is in red in the merged images. Scale bar is 50 µm. **b** Bar chart shows cell cycle progression for individual MCF7 mRuby-PCNA cells over the imaging period where each line represents an individual cell. Progression through cell cycle phases is marked by different colours: G0/G1 is green, S-phase is blue, G2 is orange and

Mitosis (M) is red. **c** Graph shows phases lengths for phases where both entry and exit into a phase was included in the imaging period. **d** Correlation score between EdU proxy gene signature and G0 score, highlighting the optimal thresholds for defining cycling and G0 tumour cells. **e** Continuous G0 score in malignant cells. **f** Differential gene expression across cell cycle states. **g** Predicted cell cycle categories (UMAP) by ccAFv2 tool and their proportions across our cell cycle states (bar). **h-i** Expression of *MALAT1* **(h)** and *GLB1* **(i)**. ***$p < 0.001$, Kruskal-Wallis test. **j** Percentage of cycling, intermediate and G0 cells per patient. **k** Kaplan-Meier survival plots for relapse-free survival in ER+ (Luminal A and Luminal B), Her2+ and TNBC+ tumours from METABRIC cohort, stratified using the G0 score into quiescent (high levels of G0 arrest) and proliferating (high levels of cycling cells).

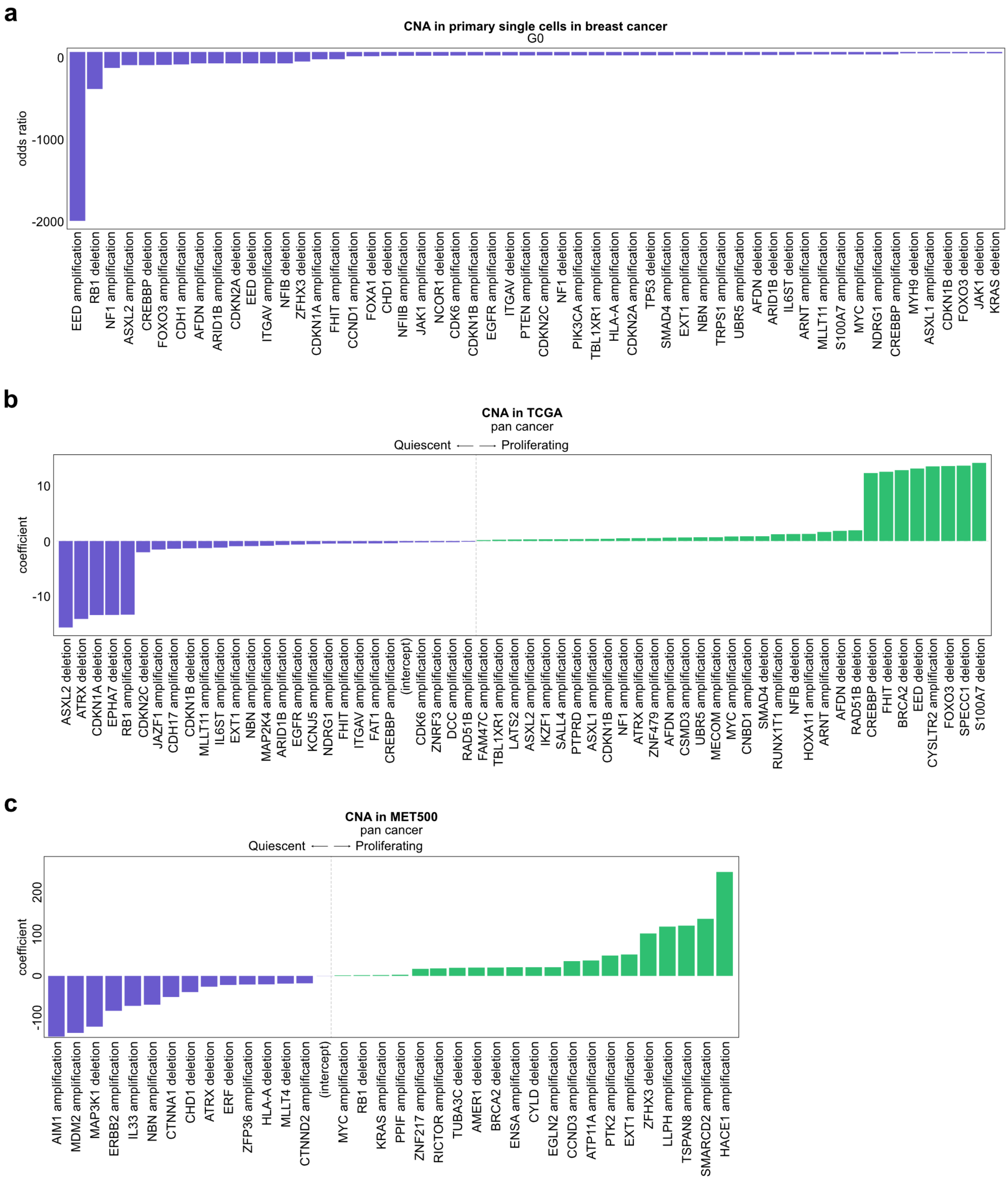

Fig. S3. Genomic alterations in cancer drivers linked with tumour cell cycle state. **a** Copy number changes in cancer drivers differentially present in G0 cells in primary single cell breast cancer cohorts, calculated using Fisher's exact tests. **b-c** Genomic changes in cancer drivers linked with slow cycling versus fast cycling TCGA primary tumours **(b)** and MET500 metastatic tumours **(c)**, inferred based on logistic regression models.

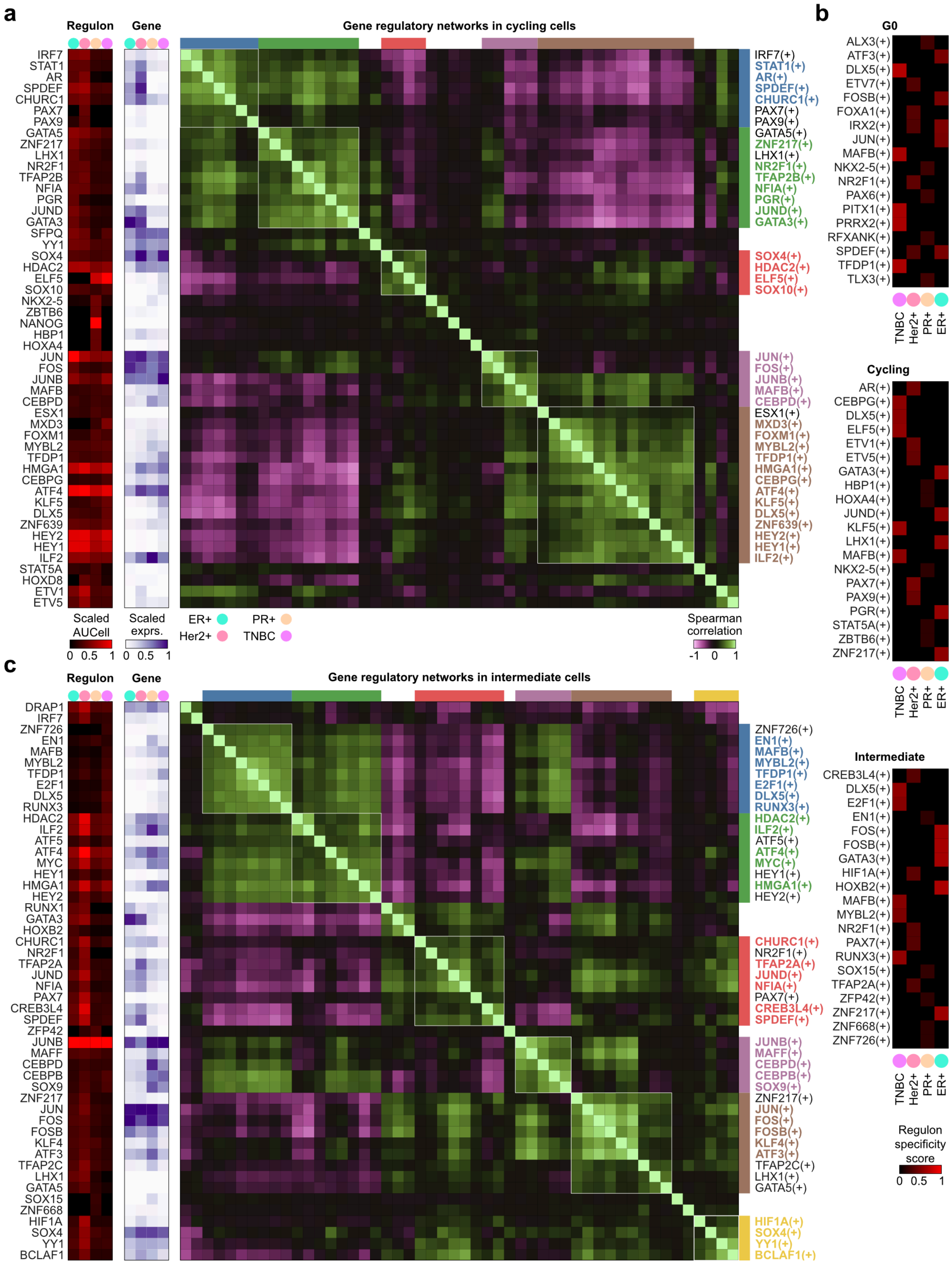

Fig. S4. Gene regulatory networks in cycling and intermediate cells. a Heat map of correlation scores for the top 50 transcription factors (TFs) in cycling cells, with modules (M1–M5) highlighted. **b** Top five TFs in each cell cycle state across cancer subtypes. **c** Heat map of correlation scores for the top 50 TFs in intermediate cells, with modules (M1–M6) highlighted.

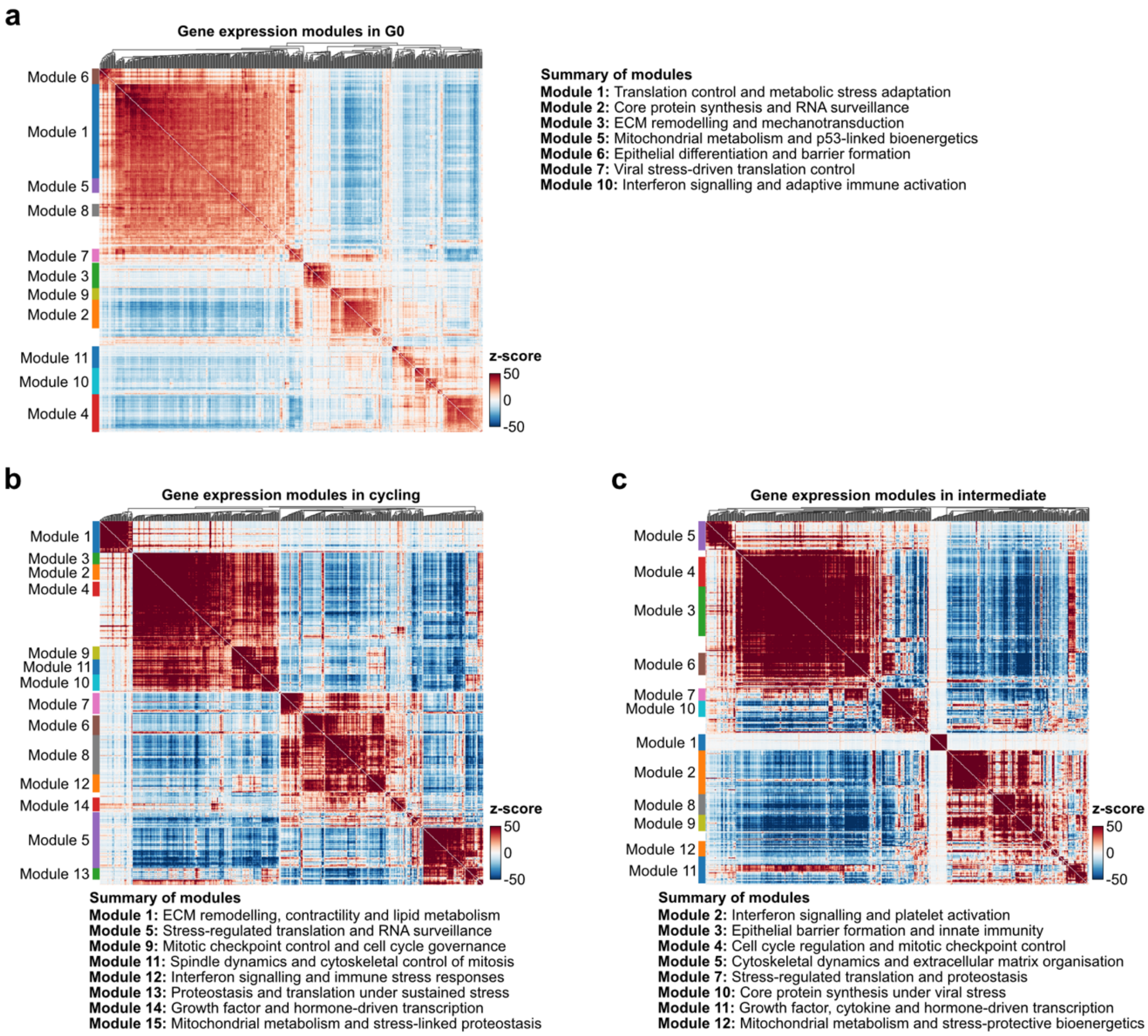

Fig. S5. Gene expression modules. a-c Heat map of gene expression modules for G0 **(a)**, cycling **(b)** and intermediate **(c)** cell states along with ReactomePA annotations.

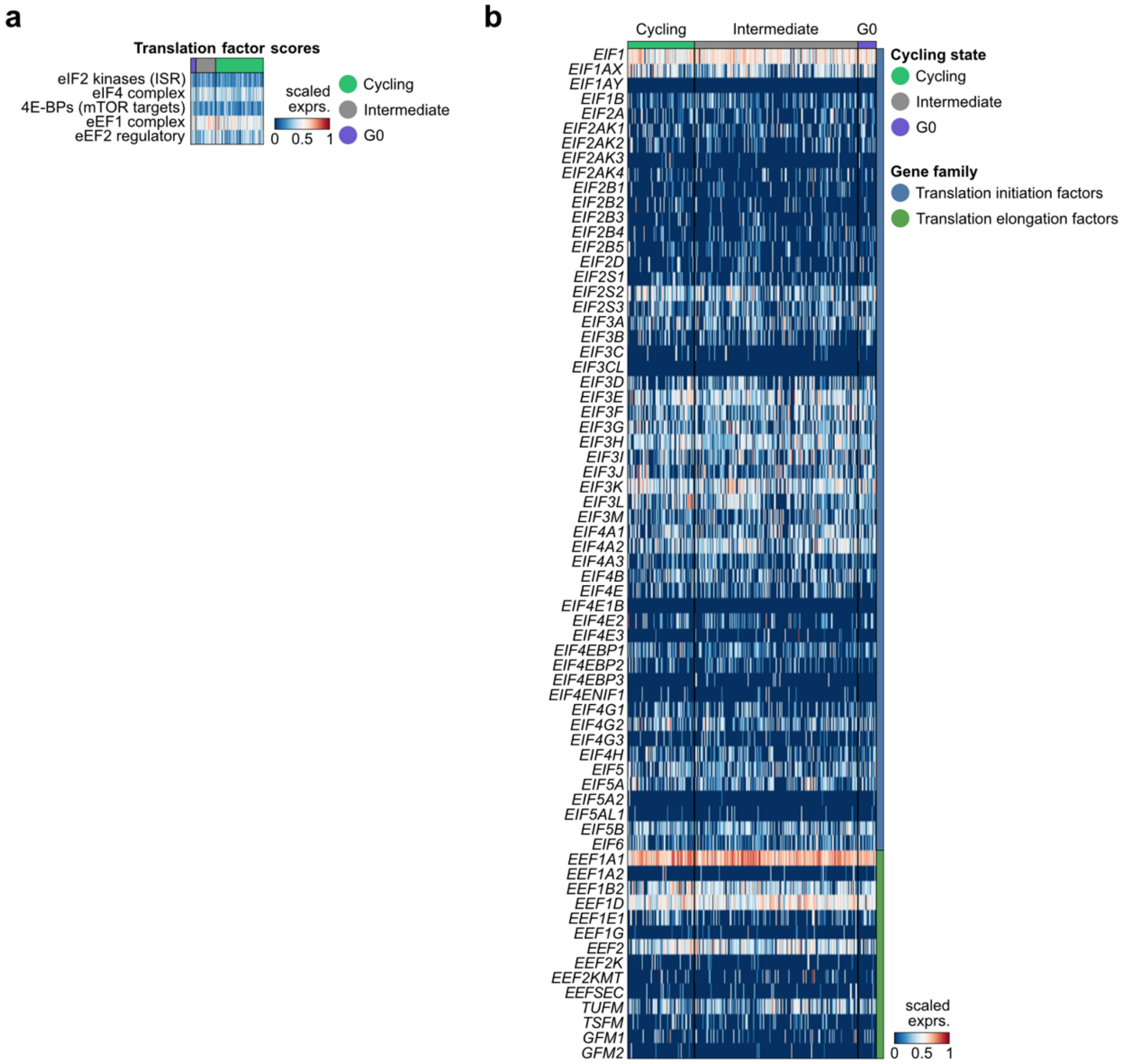

Fig. S6. Translation factors across cell cycle states. a-b Translation initiation and elongation factor enrichment **(a)** and individual gene expression **(b)** across cell states.

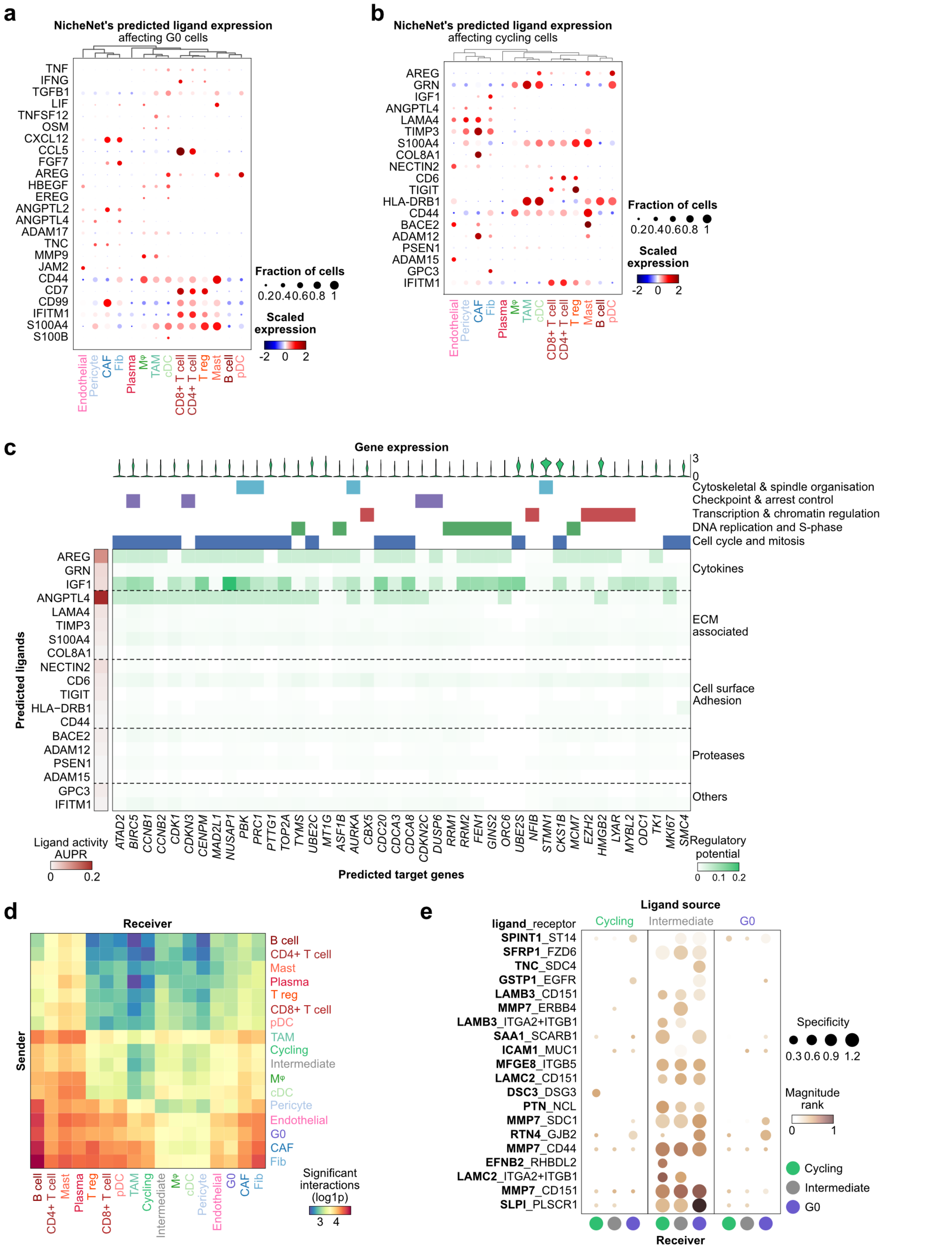

Fig. S7. Cell-cell interactions in the TME. a-b Bubble plot of predicted ligand expression in the TME affecting G0 **(a)** and cycling **(b)** cells. Rows correspond to ligands expressed by G0 cells, columns indicate the receiving cells in the TME. **c** The top 20 ligands expressed by cells in the TME and their target genes in cycling cells, as inferred by NicheNet. Rows highlight ligands on immune and stromal cells, alongside their activity depicted by a white-red colour

gradient. The ligands are grouped manually into biologically relevant categories, annotated to the right. Columns depict downstream targets in fast cycling cancer cells. Violin plots (top) show target gene expression in cycling cells. **d** Total number of ligand-receptor interactions in the TME. **e** Ligand-receptor pairs across cell cycle states, as inferred by CellPhoneDB.

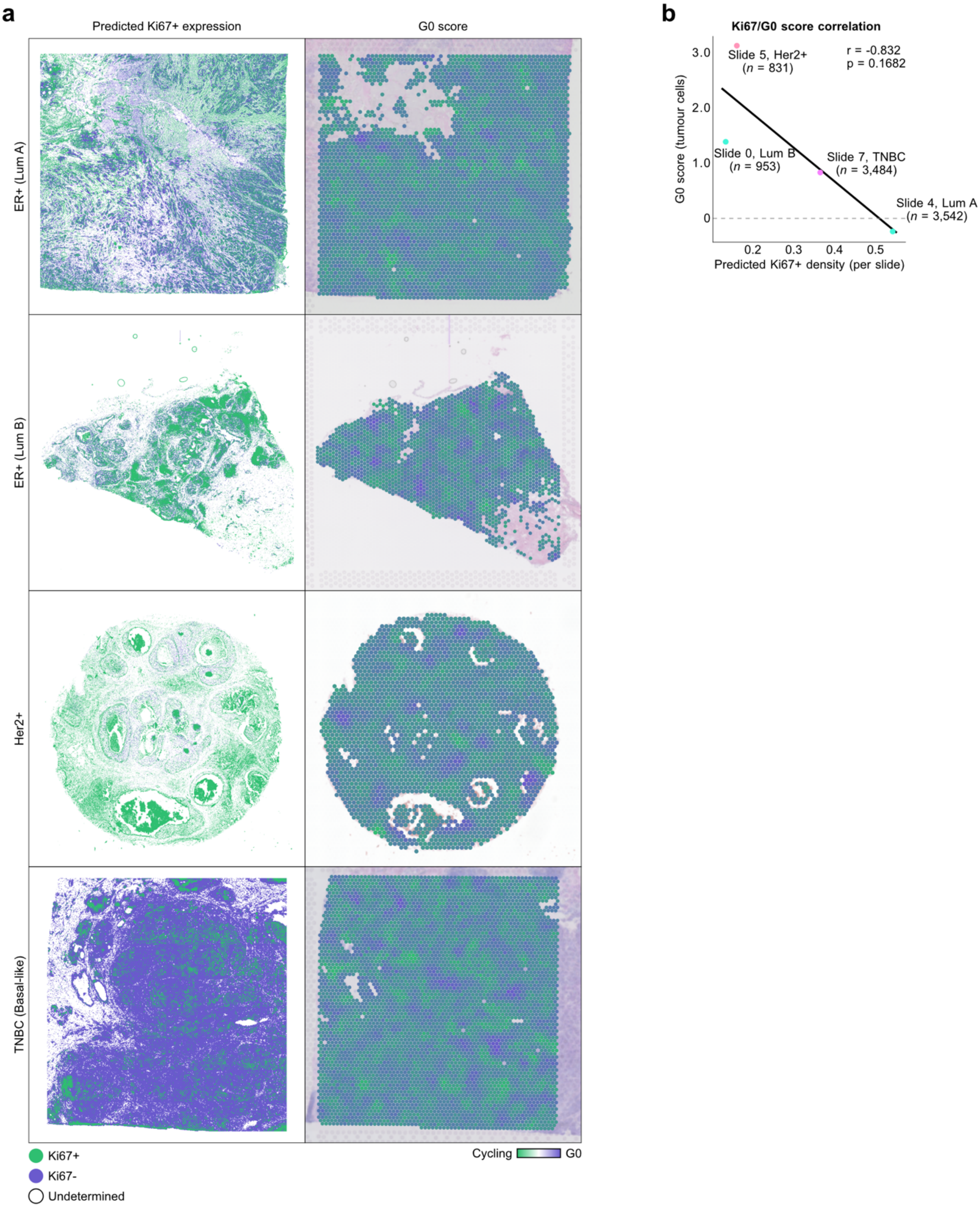

Fig. S8. Spatial characteristics and drug response. **a** Ki67 prediction from the H&E sections (left) and our G0 score across all spots (right). **b** Correlation of predicted Ki67+ and G0 scores per slide.

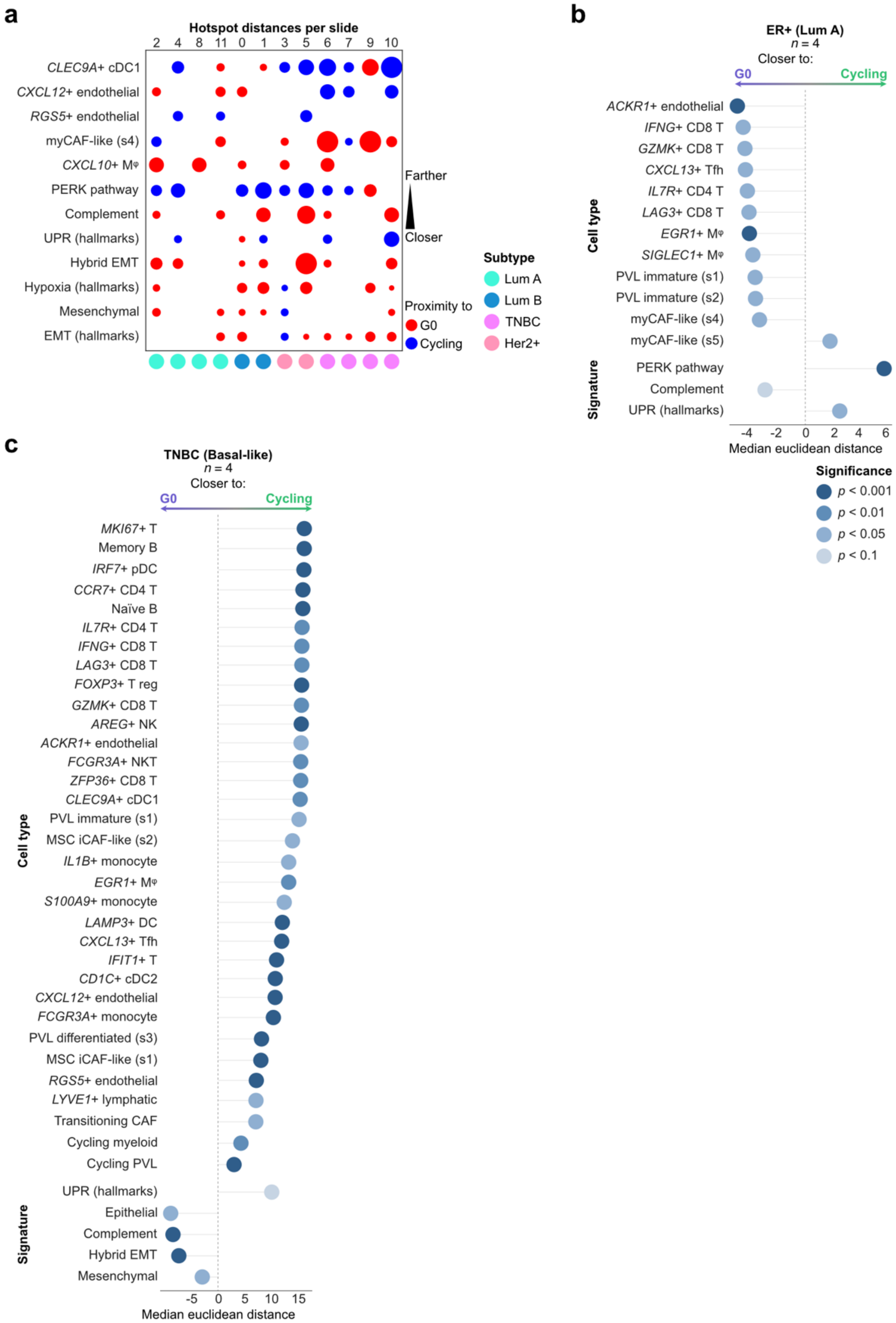

Fig. S9. Spatial TME-tumour association in breast primary tumours. a Median Euclidean distance analysis showing spatial proximity of cell types and pathway signatures to G0 versus cycling hotspots per slide. Dot colour indicates statistical significance. **b-c** Hotspot distances comparing cell types and signatures to G0 and cycling hotspots for ER+ **(b)** and TNBC patients **(c)**.

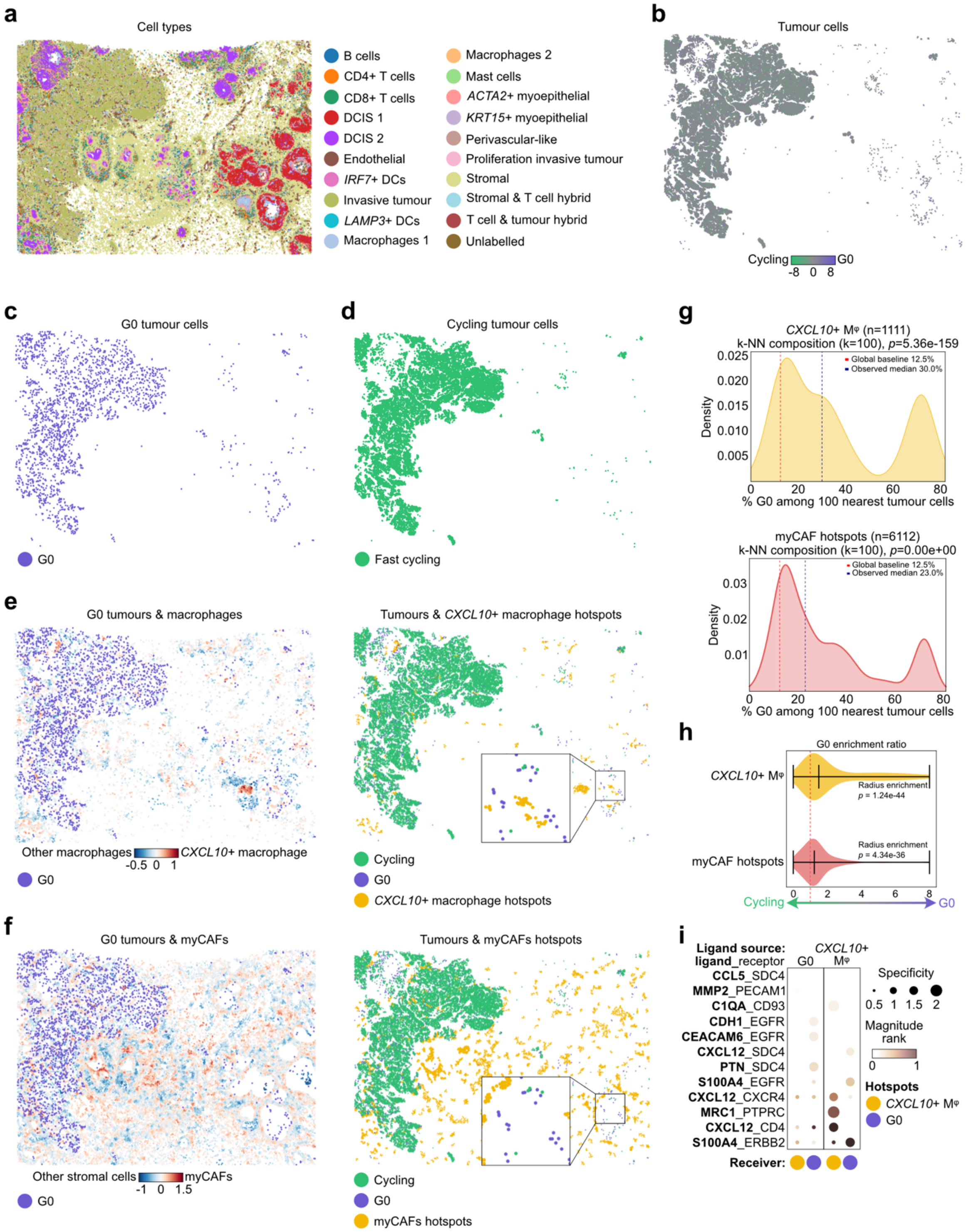

Fig. S10. Orthogonal validation of G0 cell spatial organisation using Xenium spatial transcriptomics. a Spatial map of cell type annotations in Xenium breast cancer tissue section. **b** G0 score mapped to tumour cells, showing spatial distribution along the cycling (green) to G0 (purple) continuum. **c-d** Spatial distribution of G0 **(c)** and cycling **(d)** tumour cells. **e** Left: G0 tumour cells (purple) overlaid with macrophage population coloured by *CXCL10+* macrophage score (blue to red scale). Right: G0 (purple) and cycling (green) tumour cells with *CXCL10+* macrophage hotspots (orange). Zoomed in area highlights proximity

between G0 and *CXCL10*+ macrophage hotspots. **f** Left: G0 tumour cells (purple) overlaid with stromal population coloured by myCAF score (blue to red scale). Right: G0 (purple) and cycling (green) tumour cells with myCAF hotspots (orange), demonstrating spatial separation from myCAFs. Zoomed in area highlights proximity between G0 and myCAF hotspots**. g** Distance distributions from G0 cells to nearest *CXCL10*+ macrophage and nearest myCAFs. **h** Violin plot comparing distances from G0 cells to nearest *CXCL10*+ macrophage or myCAF hotspots. **i** Ligand-receptor interaction analysis between G0 tumour cells and *CXCL10*+ macrophages. Dot size indicates specificity; colour intensity indicates magnitude rank.

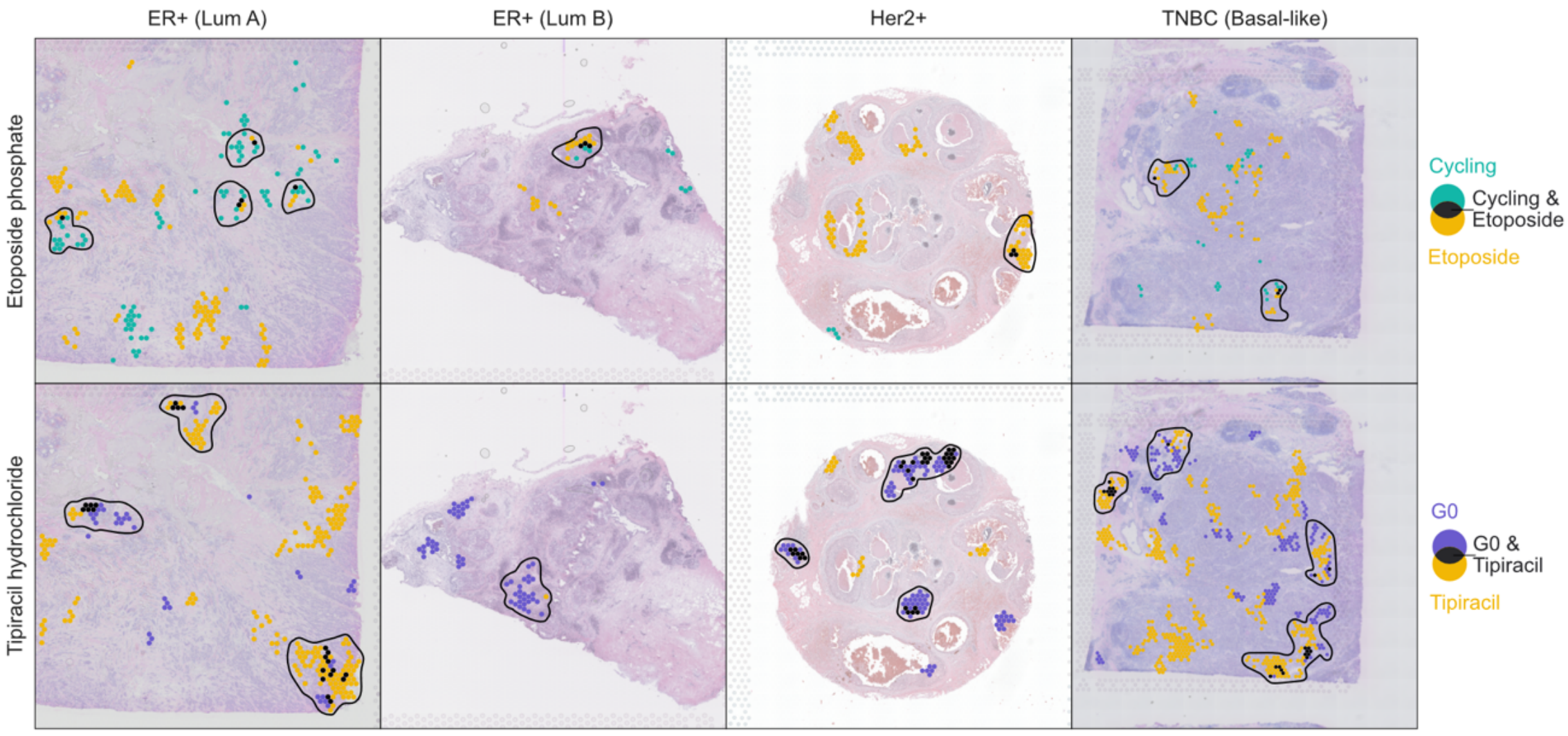

Fig. S11. Drug-effective hotspots. Drug-niche hotspots identified across breast cancer subtypes from a, illustrating areas within the tumour where the drug is predicted to be most effective and their overlap with cycling hotspots (top row, shown for etoposide phosphate) or G0 arrest hotspots (bottom row, shown for tipiracil hydrochloride).

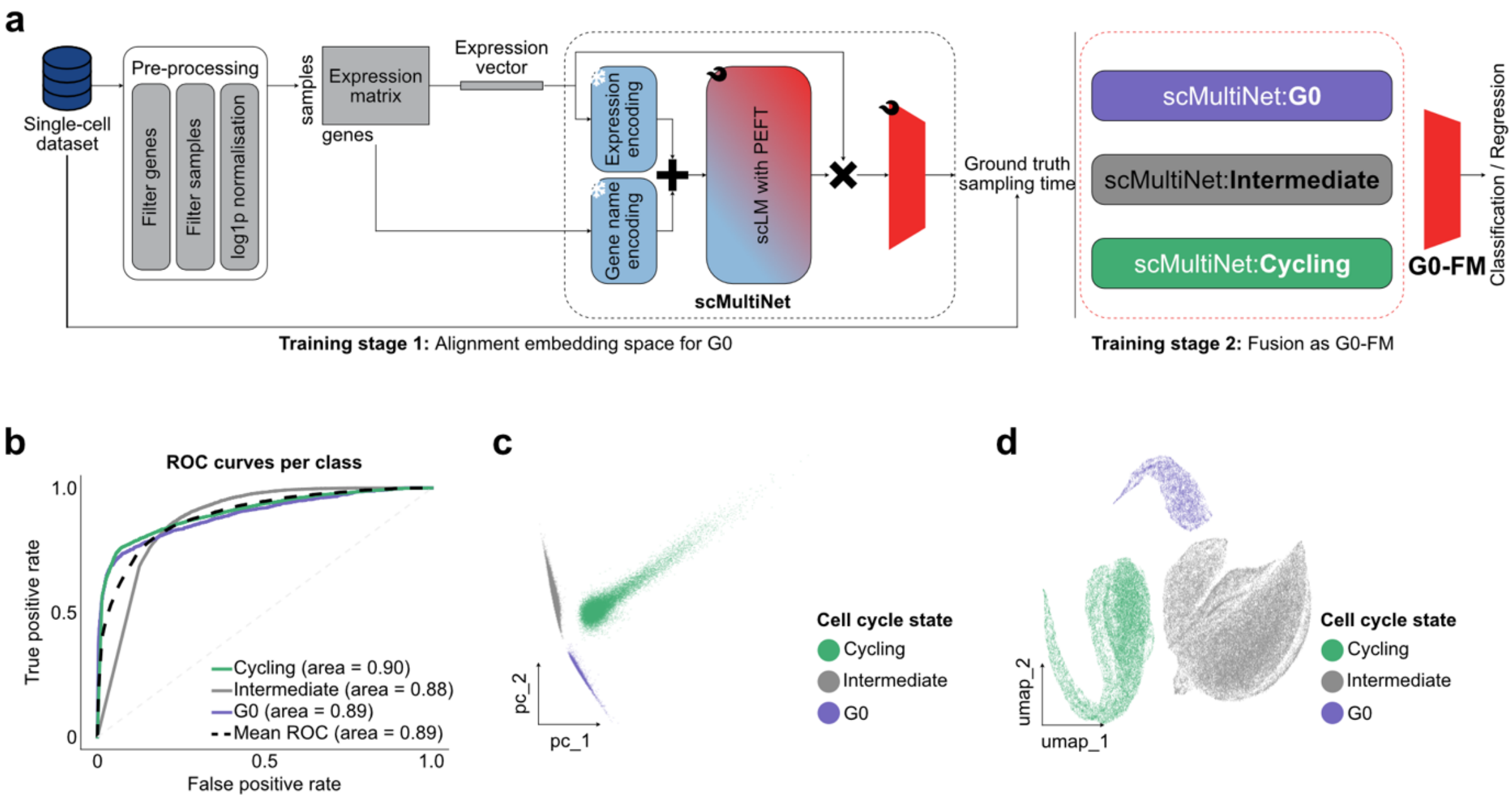

Fig. S12. G0-FM classification pipeline and performance. a G0 arrest and proliferative state classification pipeline in scRNA-seq data. The first training phase involves binary classification training for each state separately using the Parameter-Efficient Fine-Tuning (PEFT) method. This allows the original pre-trained model's feature space to better represent G0 arrest/proliferation. The final G0-FM model is obtained by fusing the three binary classification models for the individual states (G0, intermediate and cycling). **b** Receiver operating characteristic (ROC) curves per cell cycle category displaying performances of G0-FM. **c-d** PCA **(c)** and UMAP **(d)** embedding spaces of the cell cycle categories.
